# Colloidal quantum dots for thermal infrared sensing and imaging

**DOI:** 10.1186/s40580-019-0178-1

**Published:** 2019-03-05

**Authors:** Shihab Bin Hafiz, Michael Scimeca, Ayaskanta Sahu, Dong-Kyun Ko

**Affiliations:** 1 Department of Electrical and Computer Engineering, New Jersey Institute of Technology, Newark, NJ 07102 USA; 2 Department of Chemical and Biomolecular Engineering, New York University, Brooklyn, NY 11201 USA

**Keywords:** Colloidal quantum dots, Optoelectronics, Thermal infrared, Photodetectors

## Abstract

Colloidal quantum dots provide a powerful materials platform to engineer optoelectronics devices, opening up new opportunities in the thermal infrared spectral regions where no other solution-processed material options exist. This mini-review collates recent research reports that push the technological envelope of colloidal quantum dot-based photodetectors toward mid- and long-wavelength infrared. We survey the synthesis and characterization of various thermal infrared colloidal quantum dots reported to date, discuss the basic theory of device operation, review the fabrication and measurement of photodetectors, and conclude with the future prospect of this emerging technology.

## Introduction

**Fig. 1:**
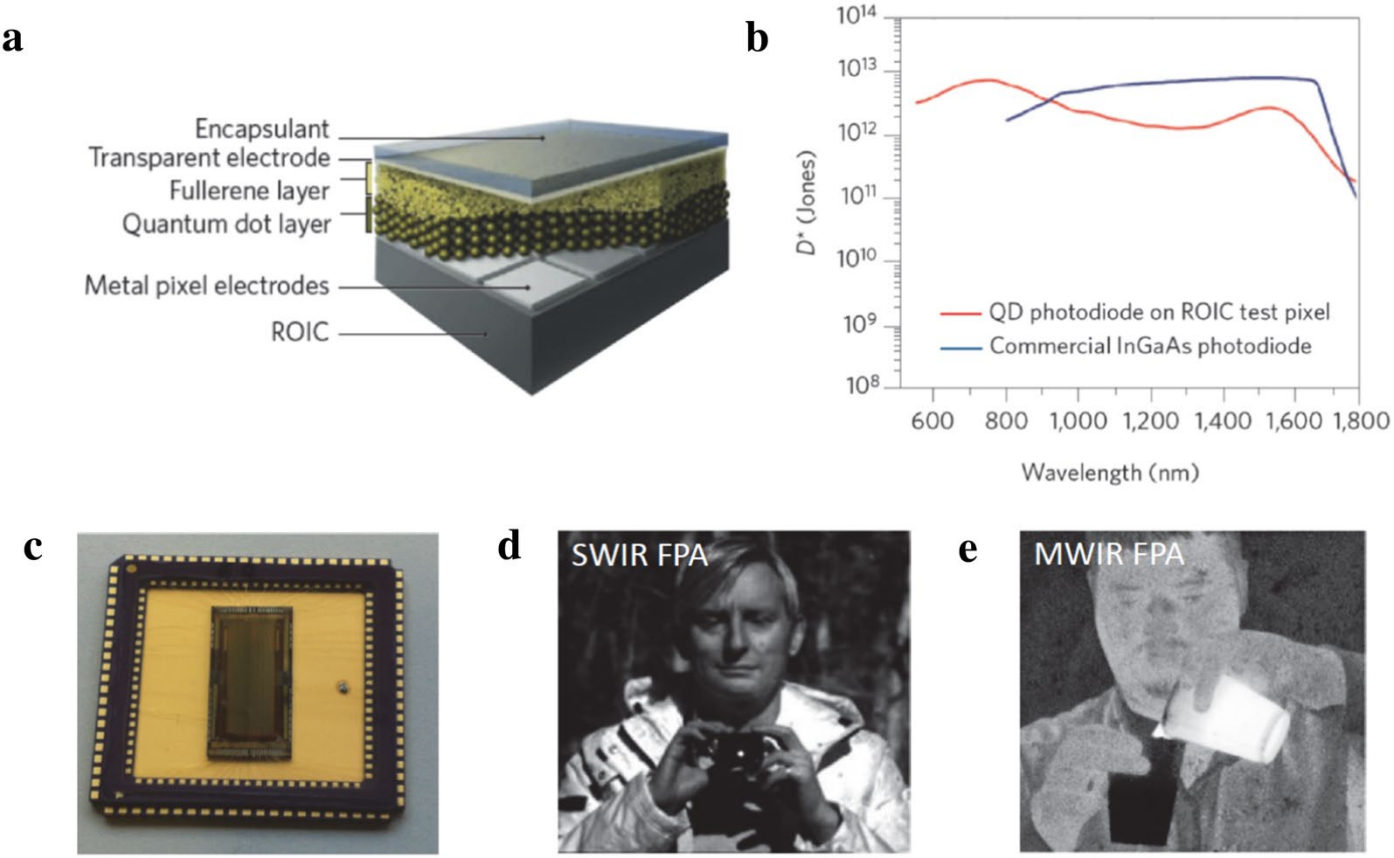
CQD-based infrared sensors and imaging FPAs. **a** Schematic illustration of CQD-based photodiode fabricated directly onto silicon ROIC. **b** Comparison of specific detectivities obtained from PbS CQD photodiode test pixel and commercial InGaAs photodiode. **c** Picture of a packaged PbS CQD-based photodiode array. **d**, **e** Images obtained from PbS CQD-based SWIR FPA and HgTe CQD-based MWIR FPA, respectively (Figures reproduced with permission from SPIE: **a**–**c** ref. [7]; **d** ref. [8]; **e** [9]. Copyright SPIE 2015, 2016)

Optoelectronics engineered from colloidal quantum dots (CQDs) benefit from greatly simplified device fabrication procedure with dramatic reduction in cost compared to traditional bulk semiconductor devices [1]. The impact that CQD-based devices would bring is expected to be significant especially in the area of infrared sensing and imaging which are currently dominated by epitaxial semiconductor technologies [2–4]. For example, HgCdTe has been a golden standard material for fabricating imaging chips, known as focal plane arrays (FPAs), operating in the mid-wavelength infrared (MWIR, 3–5 μm) and long-wavelength infrared (LWIR, 8–14 μm) spectral regions. However, despite its maturity, HgCdTe FPAs suffer from high cost of device-quality material growth and low FPA manufacturability. HgCdTe is a weakly bonded II–VI compound with high Hg vapor pressure making compositionally-uniform growth extremely difficult; in Hg_1−x_Cd_x_Te, a 0.001 variation in the composition *x* is known to drastically change the spectral response [5]. In addition, HgCdTe has a large lattice mismatch with silicon and requires high processing temperatures making it incompatible with silicon readout integrated circuits (ROIC). This has forced manufacturers to fabricate FPAs from two separate wafers, one bearing photodiodes and the other containing silicon ROIC, which are physically bonded (hybridized) together via indium bumps [6]. The complexity of multiple production steps simultaneously reduces yield and increases overall cost. Infrared CQD-based photodetector can provide attractive solutions to overcome these limitations. Spectral response can be tuned simply by adjusting the CQD size and monolithic fabrication of FPA can be readily achieved via solution-processing of CQDs directly onto ROICs at the wafer-scale (Fig. 1a) [7]. From a performance standpoint, short-wavelength infrared (SWIR, 1–2.7 μm) photodiodes based on PbS CQD have achieved high detectivities of > 10^12^ Jones at room temperature which is a detector performance comparable to commercial InGaAs detectors (Fig. 1b). Recent demonstration of low-cost SWIR and MWIR imaging (Fig. 1c, d) [8, 9] have heightened the interest in this new class of CQD-based FPAs and it is envisioned that the successful implementation of infrared CQD photodetector technology may parallel the broad impact brought by low-cost complementary metal–oxide–semiconductor (CMOS) visible cameras that are ubiquitously used today.

This mini-review focuses on emerging MWIR and LWIR CQDs and devices. For spectral regions from near-infrared (NIR) to SWIR, readers are suggested to refer to Refs. [10, 11]. The technological advantage of extending the spectral response toward longer thermal infrared lies in the fact that detectors do not require an illumination source for imaging (passive imaging) and have the ability to see through airborne obscurants such as smoke, mist, and fog [12]. LWIR imagers are particularly effective in detecting the human body as our body emission has its Plank distribution maximum around 9 μm. These make thermal infrared detectors highly suited for first-responder and search-and-rescue, night driving, machine vision, and poor weather surveillance applications that require capabilities unmet by visible, NIR or SWIR detectors. Other applications include industrial process control and preventive maintenance, environmental monitoring of hazardous chemicals [13], non-invasive measurements of temperature for tumor and blood flow [14], as well as free-space optical communications [15].

## Colloidal synthesis and characterization of thermal infrared colloidal quantum dots

The synthesis of CQDs absorbing in the MWIR range started via an aqueous reaction protocol but it was quickly found that size control in polar solvents is difficult due to aggregation and shape control limitations [16]. Therefore, organic syntheses were developed. HgTe CQDs have been the most widely studied material for MWIR absorption thus far but there are also parallel studies [17] on HgSe, HgS, and Ag_2_Se CQDs. The progress in colloidal synthesis of MWIR-absorbing CQDs are summarized in Table 1.

**Table 1:** Summary of MWIR-absorbing CQD syntheses

Material	Precursor	Ligands	Solvent	Reaction conditions (°C)	Particle size (nm)	Absorption (µm)	Comments
HgTe [19]	C_4_H_6_O_4_Hg/TOP:Te	DDT	Butanol	0 to 90	7–11 ± 3	3–5	Aggregated
HgTe [20]	HgCl_2_/TOP:Te	DDT	Oleylamine	60 to 100	5–15 ± 2	1.3–5	Non-aggregated
HgTe [31]	HgCl_2_/TOP:Te	DDT/DDAB	ODA/Oleylamine	60 to 120	5–20	3–8	Post synthesis re-growth
HgTe [39]	HgCl_2_/TMS:Te	Oleylamine	Oleylamine	60 to 100	5–12	1.5–6	Non-thiol ligands
HgTe [40]	HgX_2_ (X = Cl, Br, or I)/TOP:Te	Oleylamine	Oleylamine	120 to 340	5–200	2–65	Largest absorption range
HgSe [44]	HgCl_2_/selenourea	DDT/TOP	Oleylamine	110	5–7	3–5	Initial study
HgSe [42]	Hg oleate/SeS_2_	DDT	Oleylamine	60 to 110	6–12	3–20	Extended to 10 g yield
HgSe [40]	HgX_2_ (X = Cl, Br, or I)/NaBH_4_ reduced Se	Oleylamine	Oleylamine	120 to 340	5–200	2–65	Largest absorption range
HgS [53]	HgCl_2_/thioacetamide	DDT/Oleylamine	Oleylamine	30	4–14	3–5	Post synthesis re-growth
HgS [16]	HgCl_2_/(NH_4_)_2_S	DDT/Oleylamine	Oleylamine/TOP	80	3–15	3–10	Immiscible solvents
HgS [40]	HgX_2_ (X = Cl, Br, or I)/elemental S	Oleylamine	Oleylamine	120 to 340	5–200	2–65	Largest absorption range
Ag_2_Se [57]	AgNO_3_/TOP:Se	Oleylamine/TOP	OA, ODA, ODE	160	3–8	3–5	Monodisperse particles
Ag_2_Se [56]	AgNO_3_ or AgCl/TOP:Se	Oleylamine/TOP	Oleylamine, TOPO	160	3–10	1–7	Largest absorption range

### HgTe CQDs

**Fig. 2:**
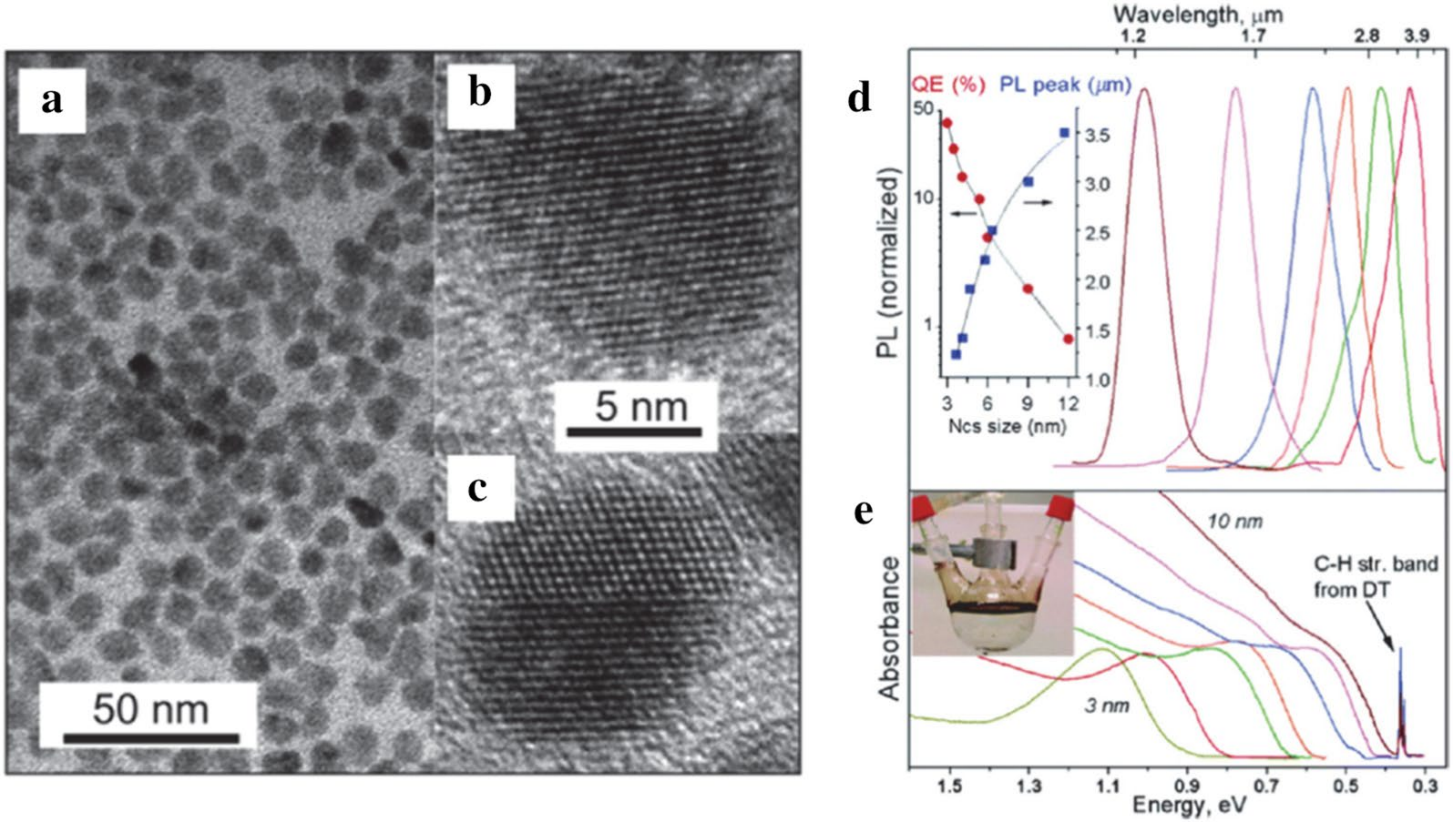
Aqueous synthesis and characterization of HgTe CQDs. **a** Low resolution TEM images showing 8 nm HgTe CQDs and **b**, **c** HRTEM images of an individual HgTe CQD. **d** Absorbance and **e** PL data for HgTe CQDs with sizes varying from 3 to 10 nm. As size increases, the absorbance edge pushes to lower energy. For the 10 nm particles, the absorbance and PL cross into the MWIR regime. The sharp peak at 0.35 eV is attributed to ligand absorption (Images reproduced with permission from ref. [18]. Copyright 2006 by American Chemical Society)

HgTe CQDs were the first material to be reported to exhibit optical absorption and photoconductive response in the MWIR and LWIR. In 2006, Kovalenko et al. synthesized these particles in an aqueous based medium. These CQDs showed high crystallinity along with size and shape uniformity as confirmed by transmission electron microscopy (TEM) (Fig. 2a–c) and X-ray diffraction (XRD). These CQDs were transferred to an organic medium via post synthesis ligand exchange procedure and enabled the demonstration of tunable optical absorption and photoluminescence (PL) in the MWIR, as shown in Fig. 2d, e [18].

**Fig. 3:**
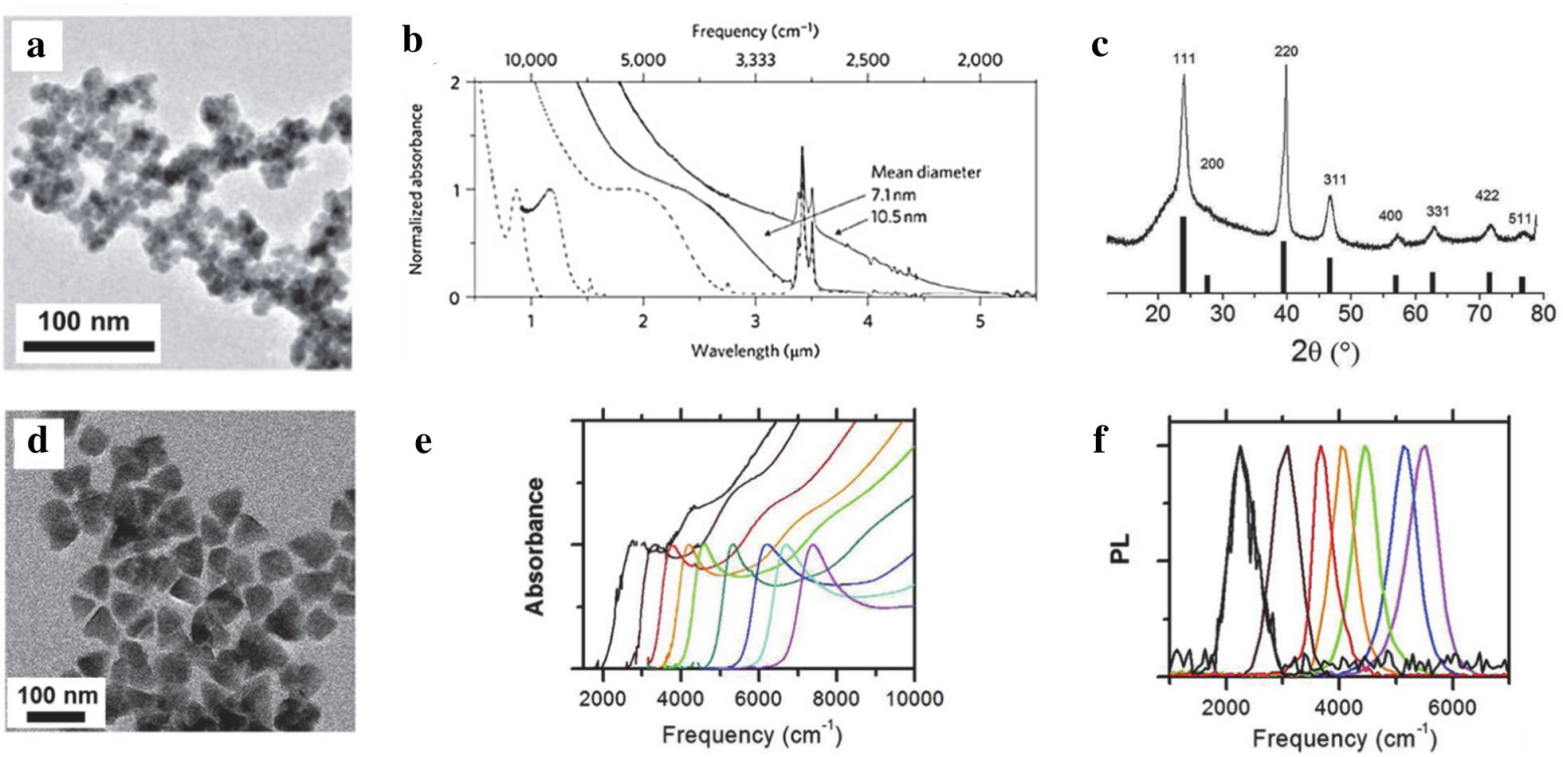
Organic synthesis and characterization of HgTe CQDs. **a**TEM image of partially aggregated HgTe CQDs. The particles are still distinguishable but the size dispersity is hard to quantify. **b** Optical absorption of HgTe CQDs from the synthesis protocol that produces aggregation prone CQDs. Images and figures reproduced with permission from ref. [19]. Copyright 2011 Springer Nature. **c** XRD data, **d** TEM image, **e** optical absorption and **f** PL emission data of the non-aggregating particles. The absorption peaks of (**e**) are much narrower and size tunable as compared to (**b**) (Images and figures reproduced with permission from ref. [20] Copyright 2011 American Chemical Society)

Briefly, the synthesis consisted of reacting Hg(ClO_4_)_2_ and H_2_Te gas at room temperature where H_2_Te gas was bubbled through in the presence of hydrophilic thiols, thioglycerol, thioglycolic acid, l-cysteine, mercaptoethanol, and mercaptoethylamine. A heat treatment is applied after the colloidal synthesis to generate larger particles sizes up to 10 nm. However, the aqueous synthesis limits the heat treatment to below 100 °C, which restricts the largest achievable particle size and thus limiting the MWIR absorption to below 3.7 µm. In 2011, Keuleyan et al. [19] succeeded in synthesizing larger HgTe particles with optical absorption in the 3–5 µm. Unlike the aqueous-based synthesis [18], this synthesis involved reacting mercury acetate(II) with Te dissolved in trioctylphosphine to obtain larger particles up to 15 nm in size. It was observed that the particles are partially aggregated according to TEM (Fig. 3a) and dynamic light scattering studies (DLS) but the particles exhibit distinct MWIR absorption up to 5 µm (Fig. 3b). The XRD reveals a zinc-blende crystal structure (Fig. 3c), which is identical to the bulk.

The aggregation led to direct integration of drop-cast films onto photoconductivity devices (without CQD film ligand exchange) as a first proof of concept experiment. This synthesis spurred the colloidal research community to re-investigate the potential of CQDs for MWIR detectors [21]. The Guyot Sionnest group followed up their previous work with a new synthesis using amine-complexed Hg-complexes and conducted the reaction at low temperature to control reaction rates. They obtained fairly monodisperse particles with a standard deviation of just 2 nm for 13 nm particles [20]. The particle size is tuned by injection temperature and growth time where the largest particles grow at over 100 °C and the smallest grow at 60 °C until the growth is terminated at various times between 1 and 90 min via a quenching solution of dodecanethiol (DDT) in tetrachloroethylene (TCE). This reaction mechanism described in detail by Howes et al. [22] leads to a narrow size distribution of HgTe particles. The particles are in a zinc-blende structure with TEM displaying particle shapes varying from triangles to distorted parallelograms to tetrahedra (Fig. 3c, d). By varying the particle size from 5 to 15 nm, the entire MWIR absorption range is covered. The absorption edge is greatly sharpened as compared to the partially aggregated particles from the prior synthesis as shown in Fig. 3e. A sharp PL peak is also observed with a full width half maximum (FWHM) of less than 500 cm^−1^ in Fig. 3f. A separate in-depth PL study was also performed using HgTe particles from this synthesis [23].

Due to the long chain amine ligands that was used to prepare monodisperse CQDs, the as-deposited CQD film now needed to be ligand-exchanged for efficient charge transport. Lhullier et al. [24–26] took the newly improved synthesis and further studied the charge carrier transport properties and optical properties after employing a ligand exchange procedure with ethanedithiol (EDT). Around the same time, Kovalenko et al. demonstrated that As_2_S_3_ can be used as an infrared transparent ligand for PbS and CdS particles [27] which led to new ligand exchange and inorganic matrix studies [28, 29]. The synthesis protocol used for these experimental works closely followed the monodisperse synthesis in late 2011 but instead of oleylamine, octadecylamine (ODA) was used. It was found that technical grade oleylamine (70% purity) led to unpredictable size distributions of particles [28]. In addition, the particles were precipitated with methanol and re-dispersed in toluene in a three-cycle centrifugation process to ensure clean particles before the ligand exchange procedure. Using this modified protocol, a 300 nm spectral resolution in the MWIR absorption range was achieved [30].

**Fig. 4:**
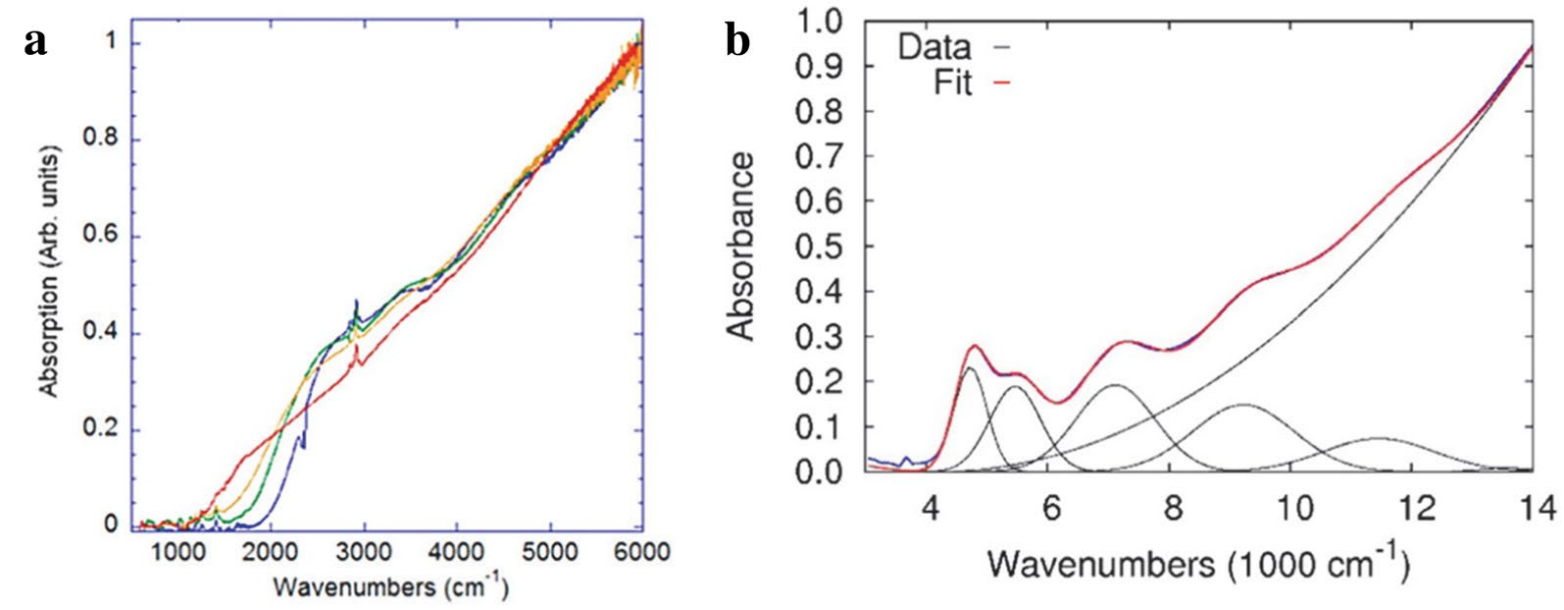
Optical characterization of large HgTe CQDs. **a** Absorption of synthesized HgTe CQDs grown to larger sizes by the dropwise addition of HgCl_2_ and TOP:Te to achieve room temperature absorption in the LWIR. **b** Absorbance spectra showing five resolvable excitonic features overlaid with a fit of six fitting functions showing as black curves (Figures reproduced with permission from ref. [31]. Copyright 2014 American Chemical Society)

The original synthesis was revisited to push the particle sizes larger to further the absorption into the LWIR [31]. Increasing reaction temperatures and durations would logically be the most straightforward way to grow larger particles. However, this was unsuccessful due to particle instability in TCE after one round of precipitation. Instead, a post reaction re-growth was initialized by adding equal amounts of HgCl_2_ and trioctylphosphine telluride (TOP:Te) dropwise in oleylamine at a temperature of 120 °C. Greater the volume of HgCl_2_ and TOP:Te added, greater was the particle size with suppressed nucleation of smaller particles. Dioctadecyldimethylammonium bromide (DDAB) was also used to assist with re-dispersion of the larger particles. HgTe particle size reached 20 nm and room temperature absorption and PL are observed past 8 µm, breaching into the LWIR for the first time exhibited in Fig. 4a.

**Fig. 5:**
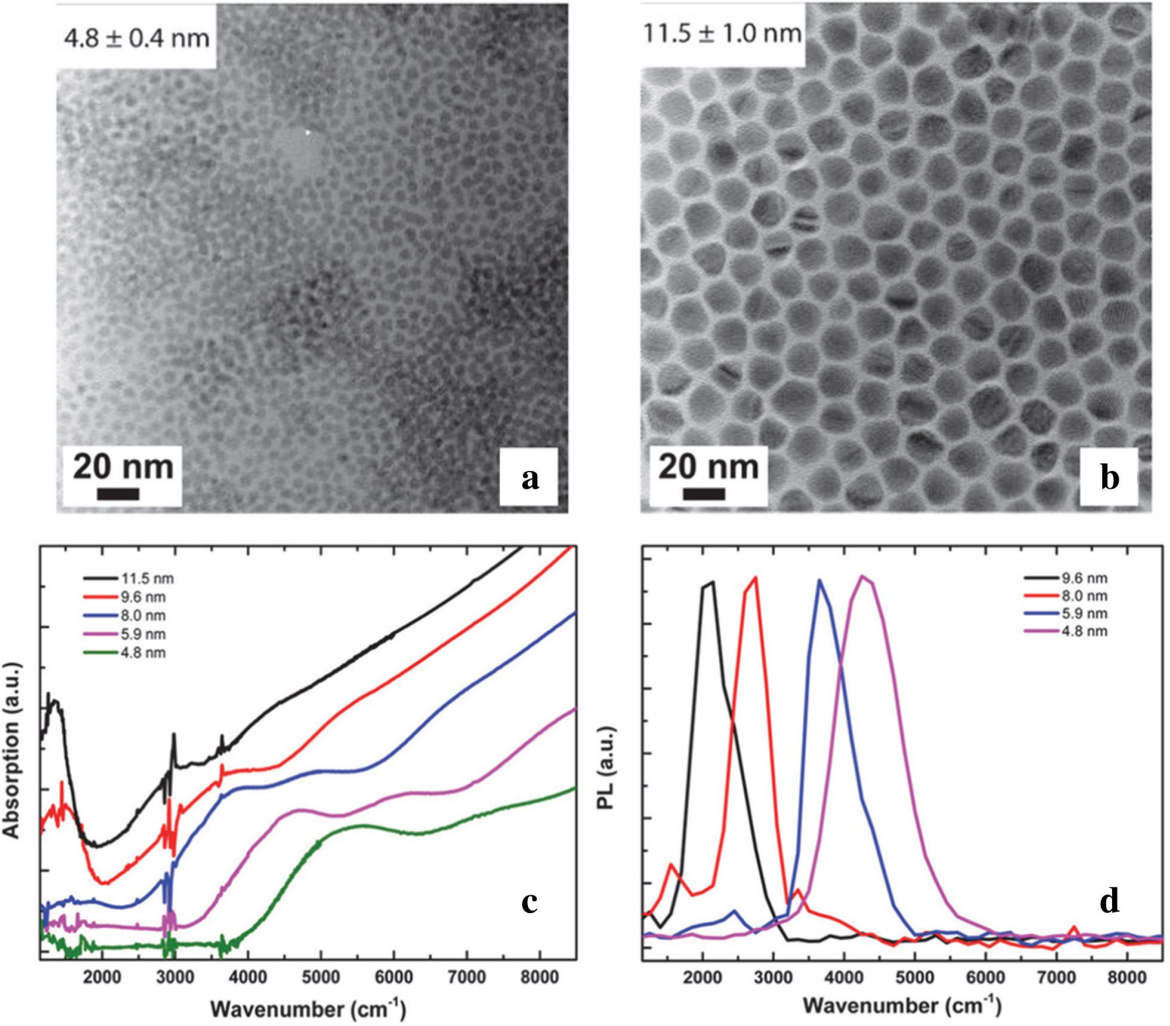
HgTe CQDs prepared via alternate precursors. **a**, **b** TEM images of HgTe CQDs synthesized using the TMS:Te precursor. The high reactivity of the TMS:Te precursor is thought to reduce faceting of the CQDs particles and exhibit a spherical shape at all sizes. **c** Absorption and **d** PL of HgTe CQDs synthesized without thiol ligands (Images reproduced with permission from ref. [39]. Copyright 2017 American Chemical Society)

In addition to pushing the particle size to 20 nm, Keuleyan et al. [31] further improved the monodispersity of the HgTe CQDs by diluting the Te precursor in oleylamine. This is hypothesized to reduce the reactivity and prevent the reaction from heavily depending on the mixing dynamics. Detailed analysis showed that the particles now exhibit five resolvable excitonic features in the absorbance spectrum as shown in Fig. 4b as compared to at most two features in particles synthesized prior to this work. TEM reveals well-defined particles that exhibit a spherical shape at small sizes and a tetrahedral shape at larger sizes. This new controllable synthesis protocol was directly used to fabricate HgTe photodetectors [32, 33] and FPAs [9, 34–36], which will be discussed in detail in the device section. The band structure and band edge dynamics of the HgTe CQDs were also investigated by reversible electrochemistry and a high bandwidth optical setup to gain a more complete insight on the electronic structure [37, 38]. Although the latest synthesis produced very narrow and size tunable MWIR absorbance and PL peaks, the strong stabilizing thiol ligands added made device fabrication difficult to process and optimize [39]. Shen et al. [39] developed a new synthesis for HgTe CQDs without thiol stabilizing ligands to facilitate photodetector fabrication. In contrast to HgTe CQDs, HgSe and HgS CQDs have greater stability even without the addition of DDT post-synthesis, which is thought to be a result of using more reactive sources of precursors of Se and S. More reactive sources of Te would logically cause concerns for CQD monodispersity but Shen et al. showed that it is not the case. NaHTe, H_2_Te, (trimethylsilyl)telluride (TMS:Te) and tributylphosphine telluride (TBP:Te) were investigated as alternative sources of Te without a TOP complex. The TOP complex is believed to act as a weak ligand for the Te sites of HgTe CQD due to steric hindrance and cause the instability of the CQDs. TMS:Te yielded the best results, which the authors attribute to trimethylsilyl being a good leaving group to avoid hindrance as a second ligand and its high reactivity with HgCl_2_. The reaction consists of HgCl_2_ dissolved in oleylamine at 100 °C where TMS:Te diluted in hexane is quickly injected to yield a black solution immediately where the specific temperature and time determine the size of the HgTe CQDs. The resulting particles are fairly monodisperse and spherical indicating that the entire surface of the nucleated CQD grows at an even rate with the new highly reactive and less bulky Te precursor as shown in Fig. 5a, b. Absorption and PL of the HgTe CQDs are size tunable throughout the entire MWIR range, as demonstrated in Fig. 5c, d.

**Fig. 6:**
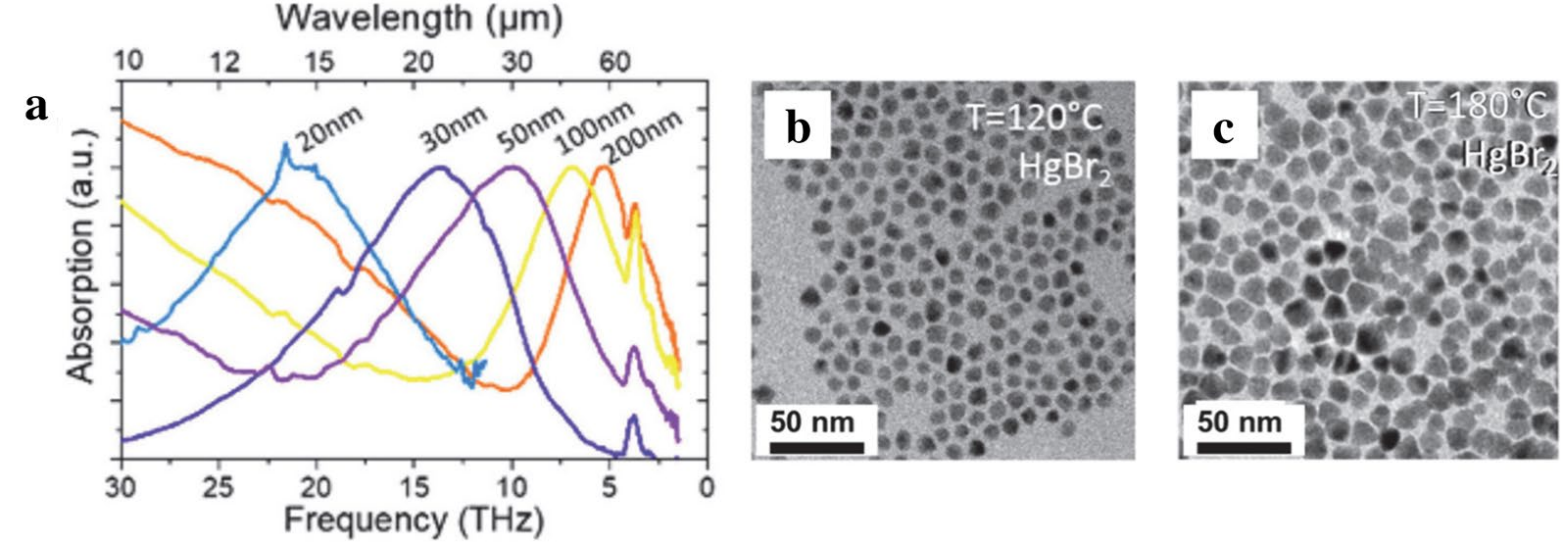
HgTe CQDs prepared via current state-of-the-art method. **a** Absorption spectra of HgTe CQDs showing the widest size-tunable absorption. The 200 nm HgTe CQDs absorb up past 60 µm. **b**, **c** TEM images of HgTe CQDs using HgBr_2_ as the Hg precursor. As the temperature increases, the particle size increases and the shapes transition from spherical to tetrahedral. The size dispersion also increases (Reproduced with permission from ref. [40]. Copyright 2018 American Chemical Society)

Goubet et al. present a synthesis that extends the absorption range of HgTe CQDs to the terahertz regime. This protocol leads to size tunable particles from 5 to 200 nm, exhibiting absorption peaks ranging from 2 to 65 µm respectively, as shown in Fig. 6a [40]. The synthesis is based on injecting HgX_2_ (X = Cl, Br, or I) and TOP:Te simultaneously into oleylamine at temperatures ranging from 120 to 340 °C depending on particle size desired. The diffraction pattern and high-resolution TEM images of the CQDs indicate well-defined zinc-blende crystal structure. Figure 6b, c show the particles are spherical for smaller sizes but the shapes and sizes start to vary as the particle size is increased. This is currently the state-of-the-art synthesis of HgTe CQDs with narrow absorption and PL peaks tunable throughout the MWIR and LWIR. A further study was reported while preparing this manuscript that details a core–shell HgTe/CdTe structure that improves thermal stability for future integration into MWIR photodetectors [41].

### HgSe CQDs

Research on HgSe CQD MWIR photodetectors is motivated by the increased resistance to oxidation of HgSe CQDs as compared to HgTe CQDs. In HgTe, the low electronegativity of Te is the cause for high oxidation sensitivity and the replacement of Te with Se is essential for progress towards air-stable photodetectors [42]. In addition, HgSe is a self-doped CQD in contrast to HgTe CQDs which are thought to be intrinsic. This opens the opportunity for both interband and intraband transitions to be explored in the case for HgSe CQDs for MWIR photodetectors [43]. MWIR absorption in this material was initially reported by Deng et al. [44] where the authors optimized a synthesis for HgSe CQDs in the 5–7 nm size range to utilize the intraband transition to absorb wavelengths from 3 to 5 µm. A typical synthesis involved using selenourea as the Se source and HgCl_2_ as the Hg source. Briefly, selenourea is dissolved in oleylamine and heated at 180 °C under nitrogen for 2 h. In parallel, HgCl_2_ is dissolved in oleylamine at 110 °C for a half hour before the selenourea solution was quickly injected. The particle size is controlled by reaction time and the reaction is quenched by a solution of a small amount of TOP and DDT in TCE. After cleaning with methanol and redispersing in TCE, the HgSe products are stable for months.

**Fig. 7:**
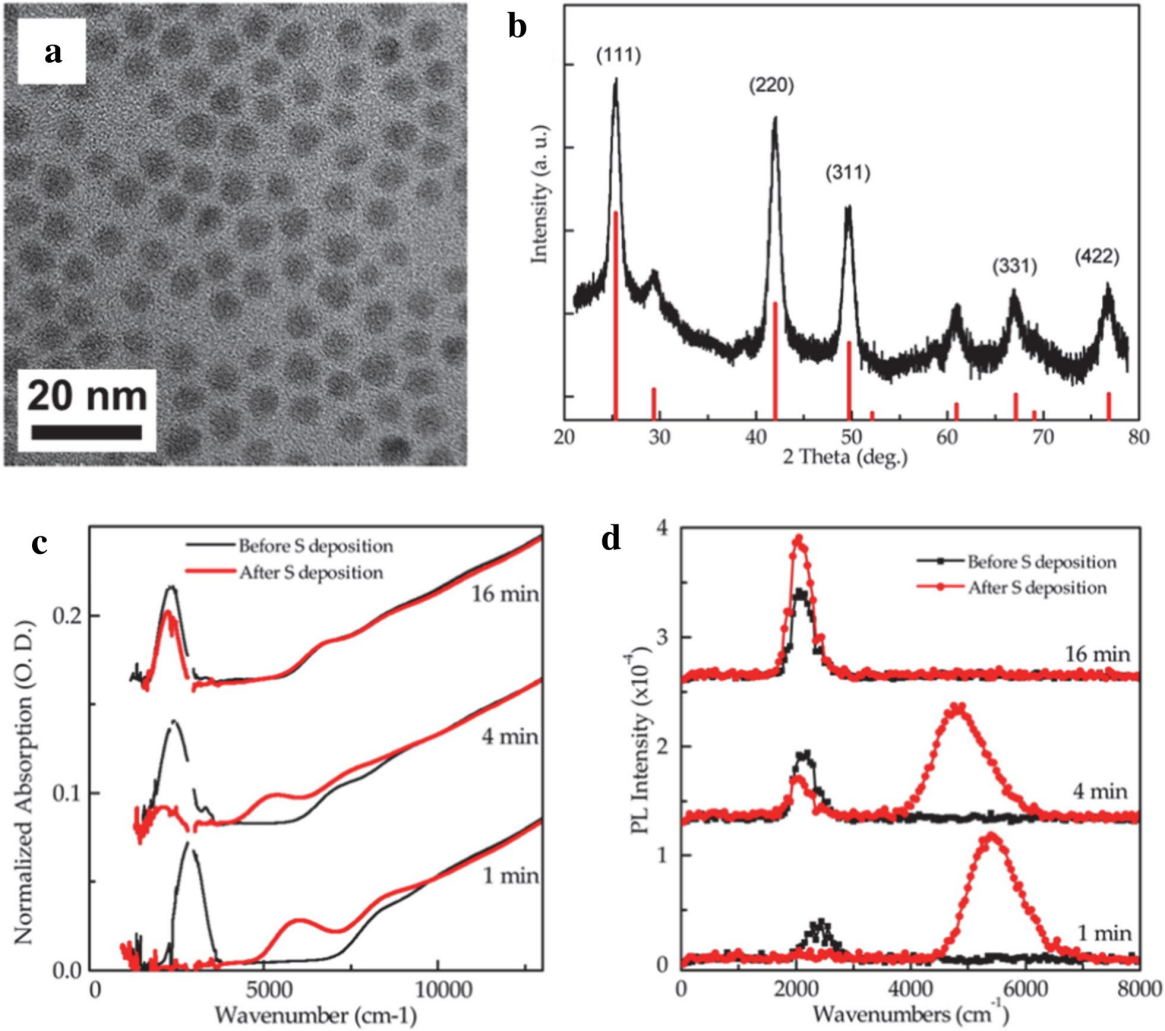
Synthesis and characterization of HgSe CQDs. **a** TEM image of HgSe CQDs using HgCl_2_ and selenourea as the precursors and **b** XRD data with peaks matching HgSe reference data. **c** Absorption and **d** PL spectra of HgSe CQDs synthesized for various amounts of time. Increased time leads to larger particles which red shifts the absorption and PL peaks due to the reduction of interband and intraband transition energies. S deposition is shown to verify the doping influence on the optical properties (Figure obtained with permission from ref. [44]. Copyright 2014 American Chemical Society)

**Fig. 8:**
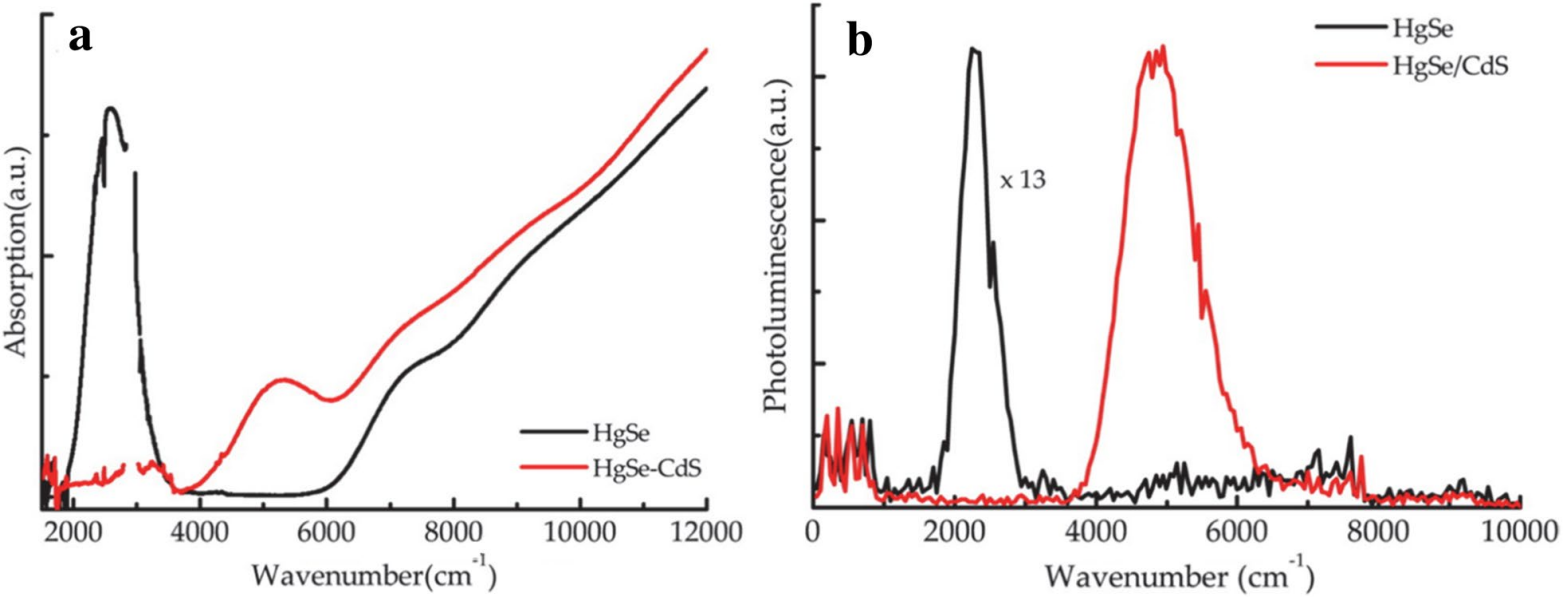
Detailed optical characterization of HgSe CQDs. **a** Absorption and **b** PL data for HgSe core only (black) and HgSe/CdS core–shell (red). The shell quenches the intraband transition but causes the formation of an interband transition which is thought to be due to the de-doping effect (Figure reproduced with permission from ref. [46]. Copyright American Chemical Society 2016)

TEM and XRD indicate that the synthesis is very robust and yields monodisperse particles while absorption and PL data consistently show two well-resolved peaks as shown in Fig. 7. The two peaks agree with a two-band k.p model and are thought to represent intraband and interband transitions. Reaction time, and therefore particle size, influences the relative intensities of the two peaks signaling that the doping level changes with particle size which is verified by intentionally modifying the doping level via sulfur deposition and observing the optical property changes (Fig. 7c, d) [44]. This is modeled using an electronic band structure schematic presented later that shows the electron occupation of conduction band states at various doping levels and is investigated by sulfur deposition, electron paramagnetic resonance (EPR) and electrochemistry measurements [37, 45]. Intraband PL with a quantum yield (QY) of 1–5 × 10^−4^ is observed in these HgSe samples which is comparable to the interband QY of HgTe samples [23]. Core–shell strategies with CdE (E = S, Se, Te) were then pursued to improve the PL of HgSe CQDs by surface passivation in a colloidal atomic layer deposition (c-ALD) process [41, 46–49]. Shell growth only occurred while using cadmium oleate as the cadmium precursor while the selection of the sulfur precursor was not as restricted [46]. Figure 8 shows that shell growth quenches the MWIR absorption and PL peaks but creates higher energy peaks at a wavenumber of 5000 cm^−1^. This phenomenon is thought to be sensitive to surface chemistry due to the change in doping and/or surface gating effects of the shell growth [46, 50]. As the original MWIR absorption and PL peaks are partially recovered after films are fabricated from solution, surface gating is claimed to be a more probable reason for this occurrence [46]. The HgSe/CdS films exhibit a threefold increase in PL as compared to HgSe core-only films.

**Fig. 9:**
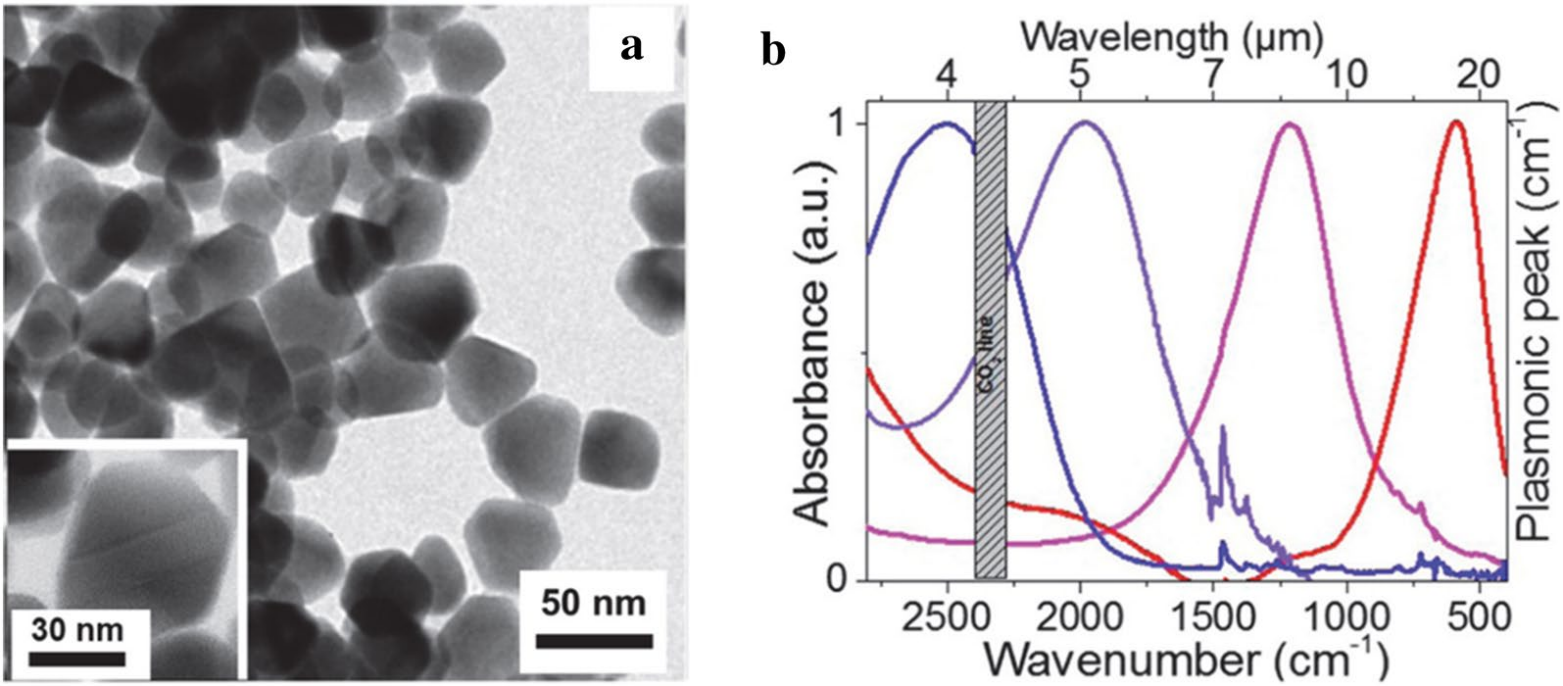
HgSe CQDs prepared via improved synthesis method. **a** TEM image of large HgSe quantum dots synthesized using Hg oleate and TOP:Se as the precursors where the shapes deviate from spheres and **b** MWIR to LWIR absorption features for the medium to large HgSe CQDs (Figures obtained with permission from ref. [42]. Copyright 2016 American Chemical Society)

In early 2016, a newly improved synthesis of HgSe was developed that can be scaled to a 10 g yield and can extend the absorption range to the THz [42]. Mercury acetate dissolved in oleic acid and selenium powder dissolved in TOP were used as the reaction components for precise control of HgSe CQDs between 6 and 12 nm in size. Oleylamine is added to the mercury oleate solution and degassed before TOP:Se is injected at temperatures ranging from 60 to 110 °C. The reaction is quenched by DDT after a certain amount of time to obtain the final desired particle size. To grow particles that absorb up to 20 µm as shown in Fig. 9, TOP:Se is replaced by SeS_2_ and the same reaction procedure is used.

**Fig. 10:**
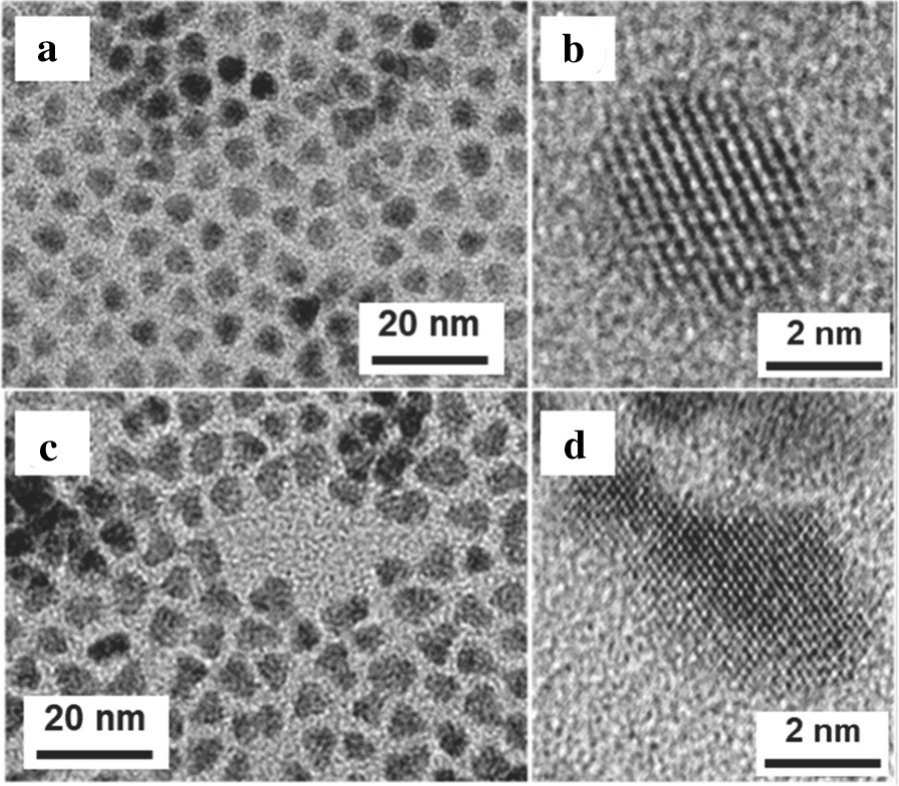
Core–shell HgSe CQDs. **a** Low resolution and **b** high resolution TEM images of the HgSe core-only particles using HgCl_2_ and TOP:Se as precursors. **c** Low resolution and **d** high resolution TEM images of the HgSe/HgTe core–shell particles showing that particles are still fairly monodisperse but have irregular non-spherical shapes that could be the result of side nucleation events (Images reproduced with permission from ref. [52]. Copyright 2018 American Chemical Society)

As-synthesized CQDs exhibit an average FWHM of 186 meV for the MWIR absorbance peak [51]. This synthesis greatly increased the attainable size range of HgSe CQDs from 4.5 to > 20 nm which inspired the study of how the electronic band structure changes with size and highlighted the semiconductor to metal transition for HgSe CQDs [43]. More recently, a new synthesis was proposed by Goubet et al. to further push the particle size up to 200 nm and the absorption edge to 65 µm for HgTe CQDs by simultaneously injecting a complex of TOP and HgX_2_ (X = Cl, Br, or I) with TOP:Te at an elevated temperature [40]. To increase the performance of intraband photodetectors, a HgSe/HgTe core–shell heterostructure was proposed [52]. HgSe zinc blende structured cores are first synthesized with a size of around 5 nm that exhibit an intraband transition at 2500 cm^−1^ and an interband transition at 7000 cm^−1^. HgI_2_ is then mixed in with the HgSe cores and TOP:Te is slowly added at a temperature from 60 to 100 °C to obtain a shell that increases the particle size to 6.4 nm and distorts the particle away from a sphere, closely resembling Janus-like nanoparticles as shown in Fig. 10. The intraband transition was preserved for the first time in a core–shell architecture [52].

### HgS CQDs

**Fig. 11:**
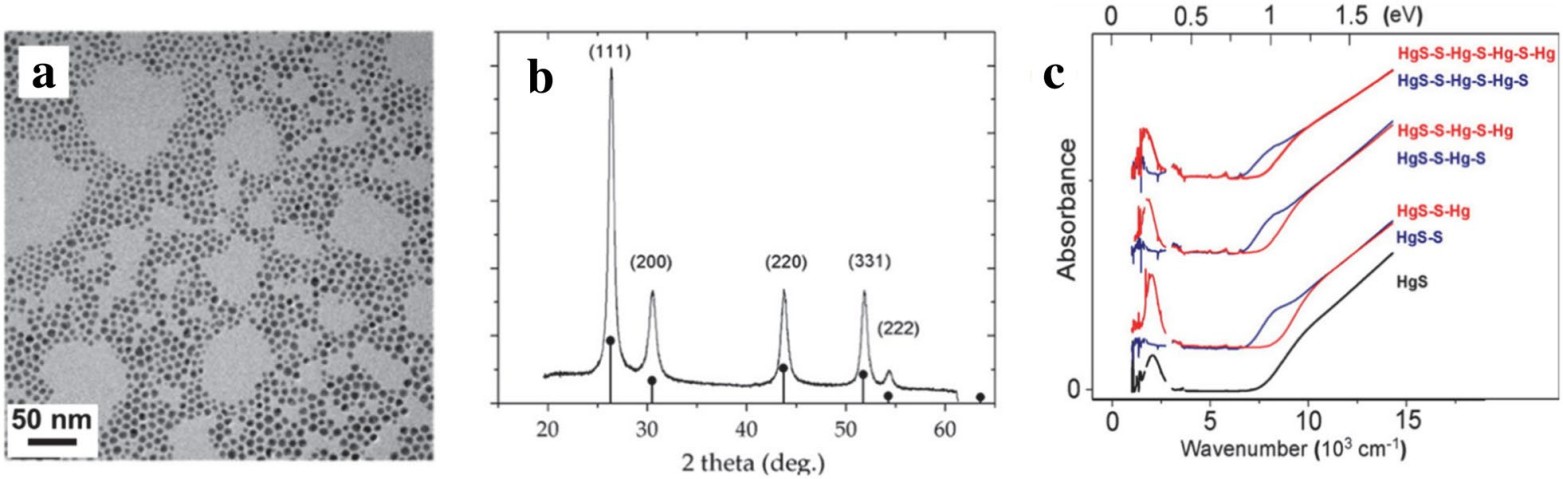
Synthesis and characterization of HgS CQDs. **a** TEM image showing HgS grown with HgCl_2_ and thioacetamide precursors, **b** XRD data indicating bulk HgS phase and **c** FTIR data revealing an interband feature at high energy and a intraband feature at lower energy (Figure reproduced with permission from ref. [53]. Copyright 2014 American Chemical Society)

In parallel to the development of HgSe CQD detectors, HgS is also a material with a unique intraband transition for use in MWIR photodetectors. Using a modified synthesis from HgTe CQDs, HgS CQDs from 4 to 14 nm were first synthesized with oleylamine ligands before replacement with DDT ligands [53]. In a later study by Yoon et al., this synthesis was slightly modified to replace DDT with excess oleylamine ligands to enable facile post synthesis ligand replacement [54]. Briefly, HgCl_2_ was dissolved in oleylamine and degassed for 1 h at 120 °C. In the meantime, the sulfur precursor was prepared from thioacetamide, bis(trimethylsilyl)sulfide (TMS)_2_S, or (NH_4_)_2_S yielding smaller to larger particles respectfully. For all precursors, a 1 M solution in oleylamine (or octadecene in later studies) was made and injected quickly into the HgCl_2_ solution where the solution immediately turned black. It was observed that the particle growth was only significant for 30 min. Further particle growth was done via a layer-by-layer addition by adding a solution of formamide, oleylamine and (NH_4_)_2_S to the HgS CQD solution in TCE. After washing to remove excess (NH_4_)_2_S, a mercury layer was added by using a solution of HgCl_2_ in formamide and washed once again in formamide, followed by characterization with TEM, XRD and FTIR, as shown in Fig. 11. These particles exhibit an intraband peak and PL emission peak in the MWIR and were also integrated in a thin film transistor (TFT) to estimate the carrier mobility [55].

**Fig. 12:**
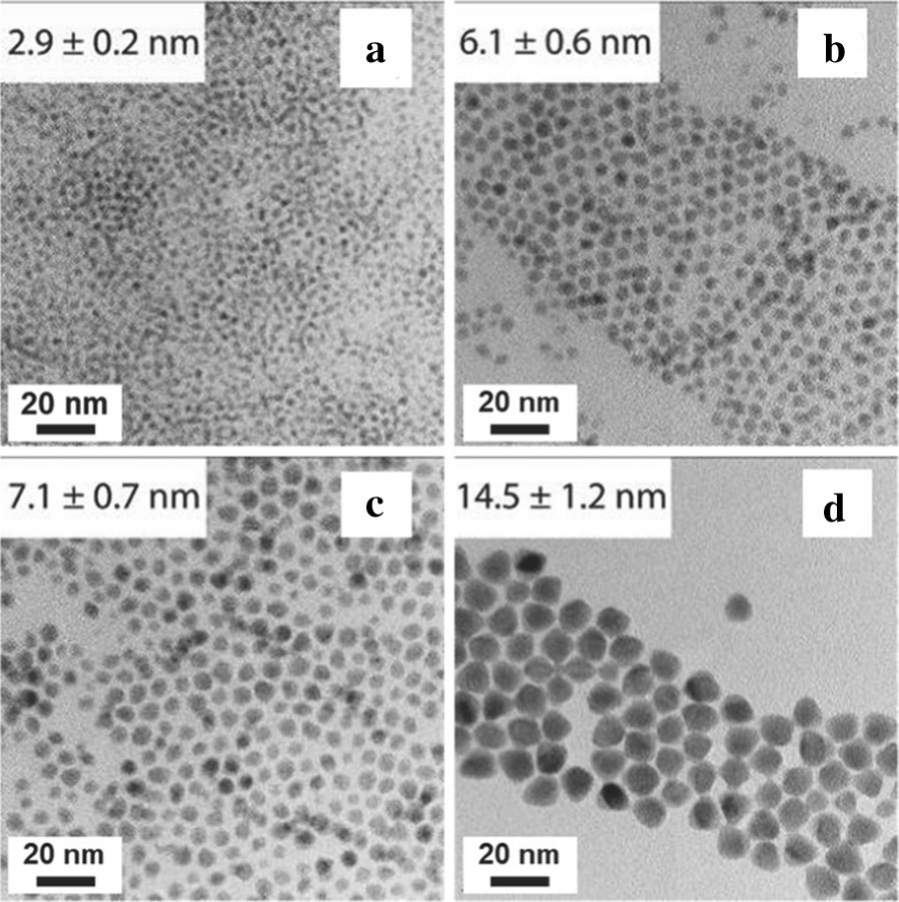
HgS CQDs prepared via alternate synthesis route. TEM images of HgS CQDs with sizes spanning from **a** 2.9 nm, **b** 6.1 nm, **c** 7.1 nm to **d** 14.5 nm (Images reproduced with permission from ref. [16]. Copyright 2016 American Chemical Society)

To focus the size distribution of HgS CQDs, a new synthesis was developed by Shen et al. [16] that used two immiscible solvents to control the reaction rate of the mercury and sulfur precursors. Remarkably, the entire synthesis is performed at room temperature in air. In a typical synthesis, mercury precursor was first prepared by mixing HgCl_2_, oleylamine, TOP and TCE at 80 °C. The sulfur precursor (NH_4_)_2_S is diluted in water and mixed with the mercury precursor described above. After 30 min, the nonpolar phase containing HgS CQDs was washed and re-dispersed in TCE. For the larger particles, DDT was needed to stabilize the particles before precipitation. The particles can be grown further via the layer by layer growth of sulfur and mercury precursors similar to the synthesis by Jeong et al. [53] and extra-large particles of 15 nm were obtained by reacting for 15 h. HgS/CdS core–shell structures were fabricated by dissolving HgS CQDs in a solution of CdCl_2_, oleylamine, TCE, diphenylamine and TOP where (NH_4_)_2_S is injected and stirred vigorously for up to 3 h before cleaning and redispersion. Precursor ratios and alternative solvent choices were extensively studied in this work, which led to various size and morphology differences in the synthesized HgS CQDs. A further study can be performed to see which combination of precursor ratio and solvent would be able to yield large particles without the need for DDT stabilization to facilitate device integration. Following this synthesis route, HgS CQDs can be synthesized in a zinc blende structure from 2.9 to 14.5 nm in size as shown in the TEM images (Fig. 12), which exhibit tunable absorption throughout the entire MWIR window.

The core–shell structure improves thermal stability but reduces electron doping thereby quenching the intraband absorption peak in the MWIR, similar to the HgSe/CdS CQDs previously reported [16]. Absolute positions of the band structure were investigated in detail via electrochemistry by Chen et al. [37]. To push particle sizes to 200 nm and absorption into the LWIR and THz level, a similar technique used for HgTe and HgSe was used for HgS as well that involves simultaneous injection of Hg:TOP and elemental sulfur into hot oleylamine [40].

### Ag_2_Se CQDs

**Fig. 13:**
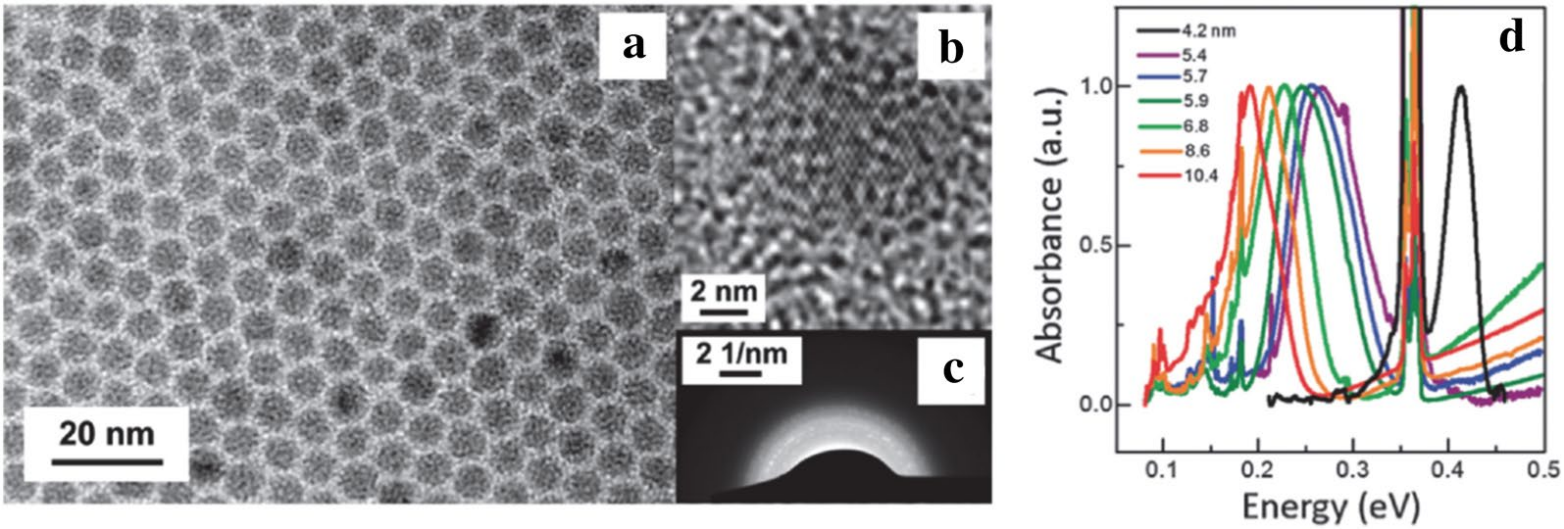
Synthesis and characterization of Ag_2_Se CQDs. **a** Low- and **b** high-resolution TEM images of the monodisperse Ag_2_Se CQDs and **c** corresponding electron diffraction. Image reproduced with permission from ref. [57]. Copyright 2011 American Chemical Society. **d** Ag_2_Se CQD FTIR data showing size tunable MWIR absorption throughout the whole MWIR window. The strong peak at 0.36 eV is attributed to the C–H stretches of the organic ligands (Figure reproduced with permission from ref. [56]. Copyright 2012 Royal Chemical Society)

More recently, Ag_2_Se CQDs (tetragonal crystal phase) have also been studied for their MWIR absorbance feature, as a viable alternative to the toxic mercury-based chalcogenides [56]. To synthesize Ag_2_Se CQDs with tunable absorption in the MWIR, AgNO_3_ and Se shot were separately dissolved in TOP to form the Ag and Se precursors [57]. Oleic acid (OA), 1-octadecylamine (ODA) and 1-octadecene (ODE) were mildly heated and degassed in a reaction vessel. As the temperature is raised to 160 °C, the TOP:Se precursor is injected first. The temperature is then further increased to a desired injection temperature and the Ag:TOP precursor is swiftly injected into the reaction vessel. The reaction time and temperature can be varied to achieve different sized particles from 3 to 8 nm in diameter. Figure 13 shows TEM images of Ag_2_Se CQDS with an average particle size of 6.5 nm and a standard deviation of 5.1%. A careful XRD analysis revealed that all CQD sizes synthesized via this method are in the tetragonal crystal phase, which is metastable in bulk.

A year later, the MWIR absorption property of tetragonal Ag_2_Se CQDs was studied in detail [56]. The synthesis was slightly modified to expand the size range to obtain particles from 2.8 to 10.4 nm in diameter while maintaining a small size standard deviation of 9%. Instead of OA, ODA and ODE, oleylamine and trioctylphosphine oxide (TOPO) were used as the reaction solvents. For particle sizes larger than 7.5 nm, AgCl in TOP was used as an alternative precursor. For the first time, Ag_2_Se CQDs were shown to exhibit absorption from 1.4 to 6.5 µm with a typical FWHM of < 75 meV, effectively covering the entire MWIR window as shown in Fig. 13b.

Further studies (Qu et al. and Park et al.) using Ag_2_Se CQDs based on this synthesis were employed to investigate the origin of the MWIR absorption and the photoconductivity of Ag_2_Se CQD devices [58, 59]. Qu et al. updated the synthesis slightly to prevent aggregation of the Ag_2_Se CQDs by also quenching the reaction with DDT ligands to stabilize the particles. An excess of Ag was reported to be the origin of excess electrons in CQDs that facilitate intraband transition in the MWIR. The history and understanding of Ag_2_Se CQD synthesis is still at a nascent stage as compared to mercury chalcogenide CQDs and can benefit greatly from further improvement in the synthesis and systematic characterization studies.

## Thermal infrared colloidal quantum dots based devices

### Theory of device operation

Colloidal quantum dots devices that form infrared sensors and imagers are largely classified into two groups: photoconductors and photodiodes. Both device structures share the same three fundamental steps of device operation. The first step is the optical absorption. Upon absorption of photons with appropriate energy, electron–hole pairs (EHPs) are generated inside the CQDs. For CQDs exhibiting interband optical transitions, electrons and holes are created at the first conduction (1S_e_) and valence energy (1S_h_) levels, respectively. For intraband CQDs, electrons and holes are created at the lowest unoccupied and highest occupied levels, respectively, inside the conduction (or valence) states. The number of photogenerated EHPs depends on the CQD film thickness, absorption cross-section of constituent CQD material and CQD packing fraction. The second step is the charge separation. The photogenerated EHPs stay in this excited state for a limited time and should be spatially separated before being annihilated through recombination (germinate recombination). In photoconductors, this is achieved through the assistance of applied bias and in photodiodes, a built-in electric field formed at the junction plays a main role in charge separation. The final step is the charge collection. The separated electrons and holes now need to be transported and collected at the respective electrodes to give rise to the photocurrent. In photoconductors, the gap between the electrodes should be small to ensure that the time it takes for photogenerated carriers to reach the electrode (transit time) is shorter than the carrier recombination (non-germinate recombination) lifetime. In photodiodes, making the width of the quasi-neutral region shorter than the carrier diffusion length is an important criterion for efficient charge extraction. From an application perspective, CQD photoconductors are frequently reported to have long-lived trapped charges. To maintain charge neutrality, the opposite charges circulate through the device many times giving rise to a photoconductive gain, making them suitable for high sensitivity sensor application in photon-starved environments [60, 61]. On the other hand, photodiodes exhibit faster response time and lower dark current, enough to be compatible with existing ROIC operation, enabling them to be used to form a 2D array for imaging at a high frame rate without motion blur or ghosting [62, 63].

**Fig. 14:**
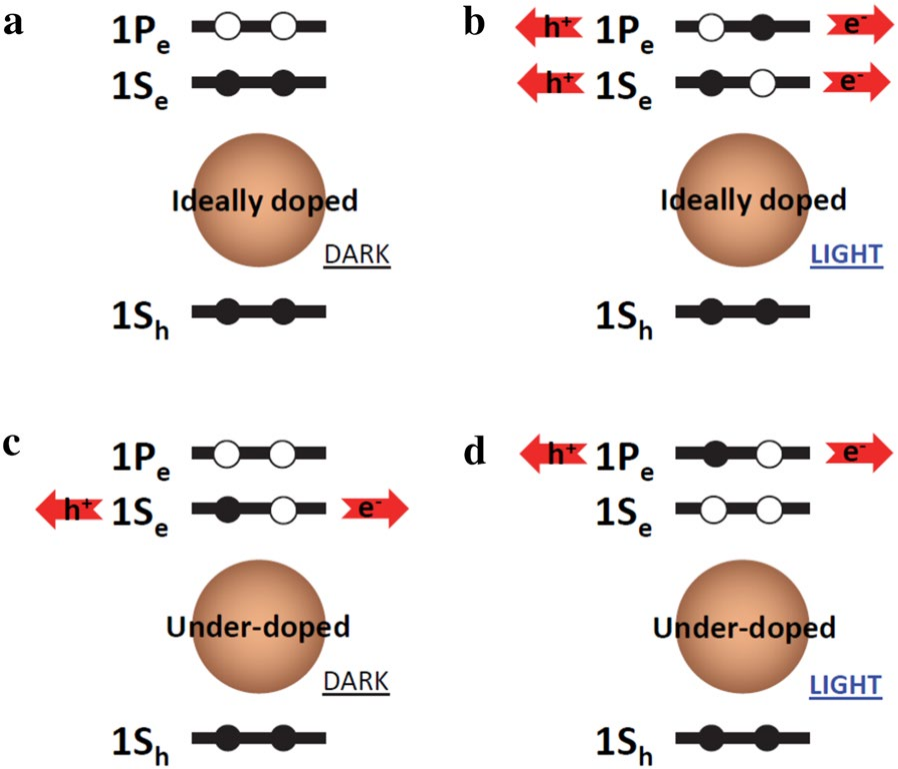
Carrier distribution and transport of intraband CQDs in ideally doped (**a**, **b**) and under-doped conditions (**c**, **d**). **a**, **c** Illustrate CQD under dark and **b**, **d** depict CQDs under illumination. 1S_h_ denotes the first valence energy level and 1S_e_ and 1P_e_ denote the first and second conduction energy levels, respectively. Red arrows represent flow of charges under bias

Under this framework, devices that rely on interband transition of CQDs is straightforward and follows the basic underlying principle of traditional semiconductor devices. Devices that utilize intraband CQDs are new and require further examination. Intraband CQD devices reported to date are all based on photoconductors and controlling the concentration of excess carriers is a key to achieving high responsivity [44]. Figure 14a depicts an intraband CQD under ideal doping condition. Under this condition, CQD film will exhibit the strongest optical absorption, as there are maximum number of electrons available to photoexcite to the second conduction energy level, and have maximum carrier lifetime, since there are minimum number of holes available in the first conduction energy level to recombine with photoexcited electrons. This condition will also lead to the lowest dark conductivity due to the scarcity of empty energy levels that electrons can hop to. Consequently, photoconductive device fabricated from this ideal CQD film will show the largest change in resistivity and hence highest responsivity under illumination (Fig. 14b). However, precise control of doping is still a challenge in CQD films and, in practice, many common ligands used for ligand exchange are observed to induce an under-doped condition, as analyzed through optical characterizations [50]. The under-doped intraband CQDs before and after illumination are illustrated in Fig. 14c, d, respectively. Under this non-ideal condition, the change in the resistivity can only be observed if the carrier mobility difference between the first (1S_e_) and second (1P_e_) conduction energy level is significant. In a similar device, known as a quantum dot infrared photodetector (QDIP) made from epitaxial quantum dot superlattices, large difference is readily achieved because electrons are bound to quantum dots under dark while electrons are photoexcited to highly mobile continuum states under illumination (typical operation) [2, 64]. In a recent study on HgSe CQDs, higher mobility was indeed observed for electrons transporting through the second 1P_e_ compared to first 1S_e_ levels [44], which was attributed to larger density of states and smaller potential barrier for transport, and enabled the first demonstration of photoconductive photodetector in the MWIR.

### HgTe CQD-based devices

#### Photoconductive devices

**Fig. 15:**
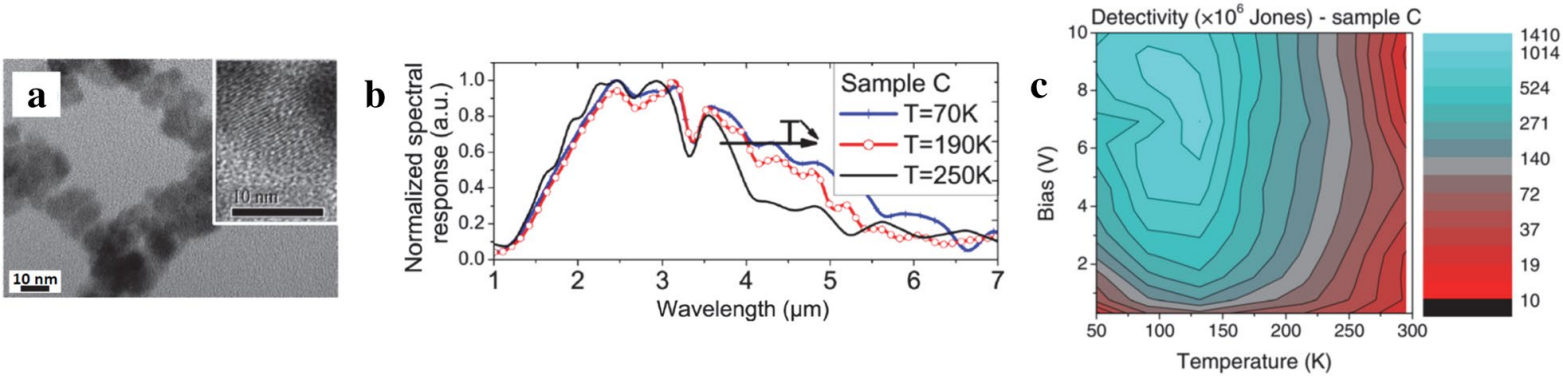
HgTe CQD-based photoconductive devices. **a** A TEM image of aggregated HgTe CQDs (6 μm absorption cut-offs) that were used to demonstrate photoconductive devices. Inset shows a high-resolution TEM image of a single CQD. **b** Normalized spectral response of these devices measured at varying temperatures. **c** The map of the specific detectivity measured as a function of temperature and applied bias (Figures reproduced from ref. [21] with permission. Copyright 2011 AIP Publishing LLC)

**Fig. 16:**
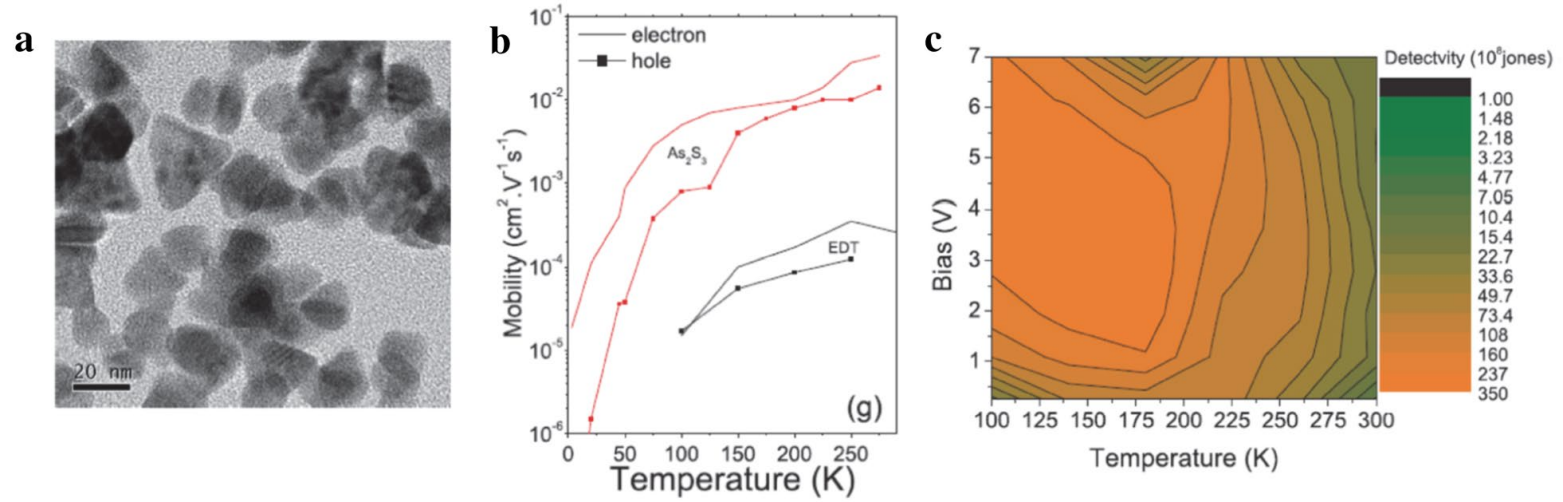
Photoconductive device based on HgTe CQDs prepared via improved synthesis. **a** A TEM image of HgTe CQDs prepared through improved synthesis method, showing reduced aggregation. **b** A plot of carrier mobility as a function of temperature obtained from CQD film ligand exchanged with inorganic As_2_S_3_-based ligand and traditional organic (ethanedithiol, EDT) ligand. **c** The map of detectivity (1 kHz) plotted against bias and temperature for 3 μm absorption cut-offs HgTe CQD device treated with As_2_S_3_-based ligand (Figures reproduced from ref. [28] with permission, Copyright 2013 American Chemical Society and from ref. [65]. Copyright 2013 John Wiley & Sons)

HgTe is a semimetal and have the prospect of covering a large portion of the infrared spectrum through size-dependent quantum confinement when they are synthesized into CQDs. Currently, HgTe CQDs are the only known colloidal nanomaterial that has been used to successfully demonstrate thermal infrared devices based on *interband* optical transition. The first MWIR photoconductive photodetector was reported by Guyot-Sionnest group [19]. The devices were fabricated by simply drop-casting HgTe CQDs on interdigitated electrodes and the as-deposited CQD film readily showed high electrical conductivity without ligand exchange due to CQD aggregation (Fig. 15). Detailed characterizations [21] have shown that CQD films exhibit a property reminiscence of an intrinsic semiconductor with high carrier mobility around 0.5 cm^2^/V s and show efficient charge separation upon optical generation, supporting the observed high responsivity. The room temperature responsivity was reported to exceed 100 mA/W (10 V bias) for HgTe CQDs having 6 μm absorption cut-off and the highest specific detectivity of 2 × 10^9^ Jones was obtained at 130 K (1 kHz) with noise observed to be dominated by 1/f noise with large Hooge’s parameter (α_H_). The response time was below 100 ns, which is faster than required for imaging application. These works were followed by improving the colloidal synthesis to obtain CQDs with better size dispersion and colloidal stability [20]. The reduced aggregation implies that the CQD films now need to be ligand exchanged for efficient carrier transport after film deposition. The HgTe CQD films ligand-exchanged with traditional organic ligands were investigated to have low carrier mobility (~ 10^−4^ cm^2^/V s) and showed degradation upon air exposure, such as the disappearance of intrinsic-like property, high dark current and slow photoresponse [28]. A proven strategy developed in CQD electronics to improve the carrier mobility is to employ inorganic-based ligands and this approach has been successfully applied to MWIR CQD photodetectors as well. HgTe CQD film ligand-exchanged with As_2_S_3_-based ligands exhibited higher mobility than organic ligands, greater air stability, and ultimately lead to 30 times improvement in the detectivity (10^10^ Jones, 1 kHz, 230 K, CQDs with 3.5 μm absorption peak) compared to that of the aggregated CQDs, as shown in Fig. 16 [28, 65]. As_2_S_3_ also have high transparency in the MWIR, making it a good candidate among other choices of inorganic ligands whereas typical organic ligands have strong vibrational modes in this wavelength range. Reducing the quantum confinement in HgTe CQDs by increasing the size would enable photodetection in the LWIR. Indeed, with the advance in colloidal synthesis, photoconductive detectors employing ~ 20 nm HgTe CQDs have successfully demonstrated photoresponse extending up to 12 μm [31].

**Fig. 17:**
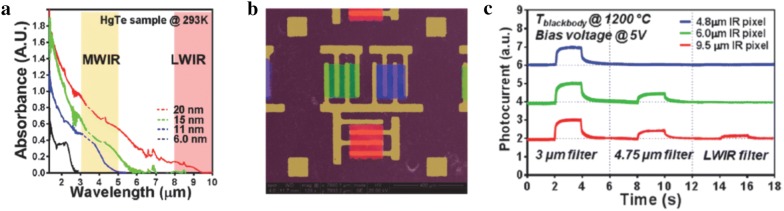
HgTe CQD-based multi-color pixel detector. **a** Optical absorbances of four different sizes of HgTe CQDs that were synthesized for three-color detector fabrication. **b** A false-colored SEMS image of three-pixel photodetector, each having different spectral response, fabricated on a single chip. **c** Photocurrent response measured from three individual pixels under incident infrared illumination with different filters (Figures reproduced from ref. [35] with permission. Copyright 2013 American Chemical Society)

**Fig. 18:**
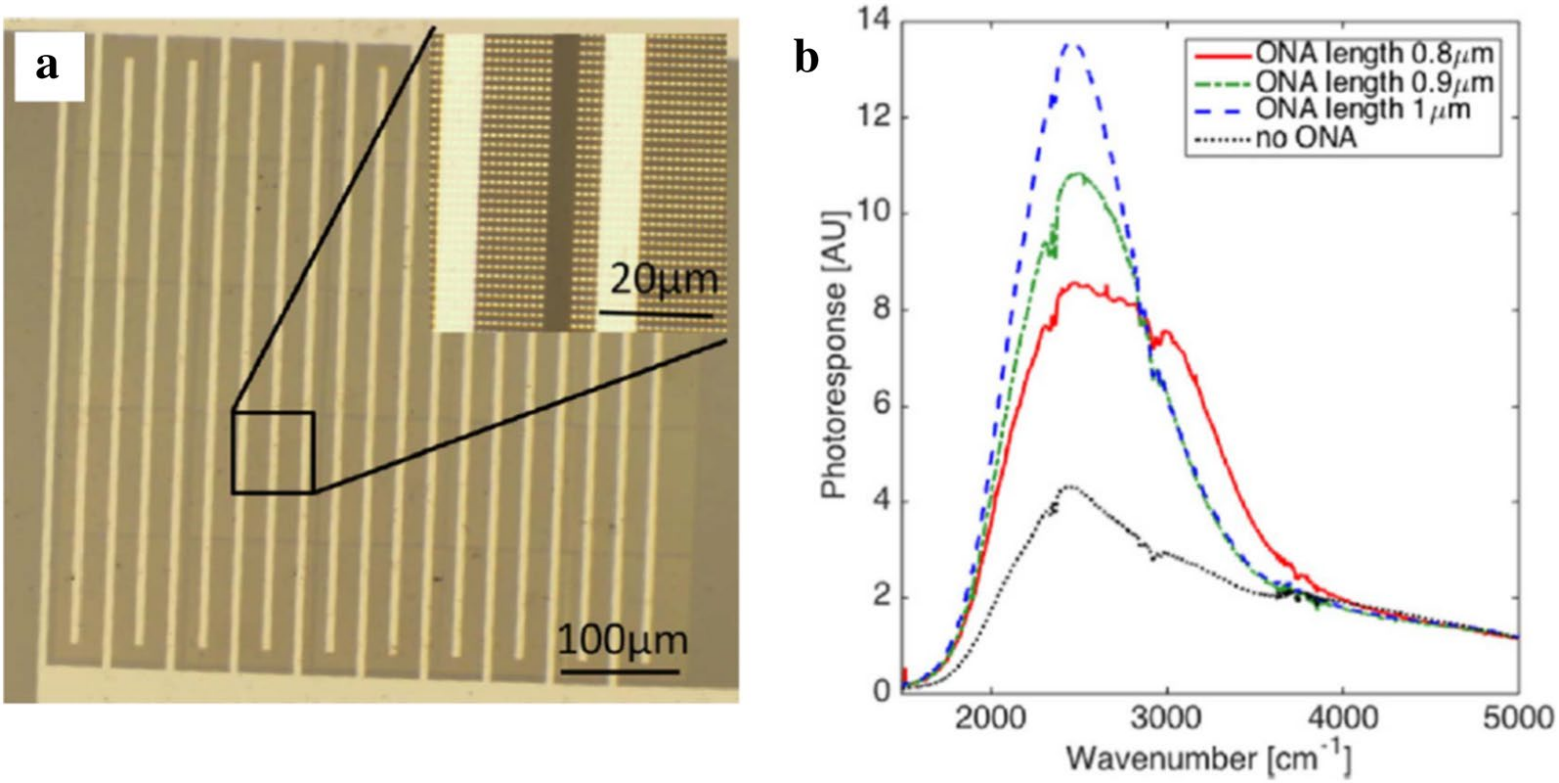
HgTe CQD-based photoconductive device integrated with optical nano-antennas. **a** Optical microscope image of gold nano-antenna array fabricated on SiO_2_ substrate. **b** The spectral photoresponse obtained from devices with and without optical nano-antenna structures at 80 K. The length of the gold nano-antenna was varied from 0.8 to 1 μm (Figures reproduced from ref. [66] with permission. Copyright 2017 AIP Publishing LLC)

By leveraging the broad spectral tunability offered by HgTe CQDs, fabrication of a 12 × 12 multicolor pixel detector on a single chip was demonstrated through poly(methyl methacrylate)-assisted transfer technique (Fig. 17). Three different sizes of HgTe CQDs having absorption cutoff at 4.8, 6, and 9.5 μm were used to fabricate 200 × 200 μm pixel elements and the responsivities of all three pixels were reported to yield tens of mA/W across each targeted infrared region under room temperature operation [35]. This was further extended to a four-color detector, operating between 2 and 5 μm, with room temperature detectivity of four pixels reaching 10^9^ Jones, which was also demonstrated on flexible polyethylene terephthalate (PET) substrates [30]. The first demonstration of MWIR imaging via HgTe CQD-based FPAs fabricated by solution-processing of CQDs on commodity ROIC were reported in 2016 [34]. Even with under-optimized CQD layer thickness and lack of anti-reflection coating, the mean detectivity at 95 K was reported to reach 10^9^ Jones with noise equivalent differential temperature (NEDT) of 2.3 K. A feasibly of imaging in the LWIR using prototype HgTe CQD FPAs was also reported recently [36]. One promising strategy to increase the photoresponse of existing CQD devices is to enhance the optical absorption using optical nano-antenna. Predesigned gold nano-antenna arrays were patterned on the substrate prior to completing the photoconductive CQD device fabrication, which resulted in 3 times increase in the photocurrent compared to the one without antennas (Fig. 18) [66].

The HgTe CQDs are of high interest in both fundamental and practical aspects as its infrared optical property arises from quantum confinement of semimetallic bulk that has inverted band structure. As the research progresses, deeper understanding of the electronic fine structures are emerging which encourages a path toward precise engineering of CQD size, shape and surface for further device improvements and optimization [67].

#### Schottky diode and p–n heterojunction diode devices

**Fig. 19:**
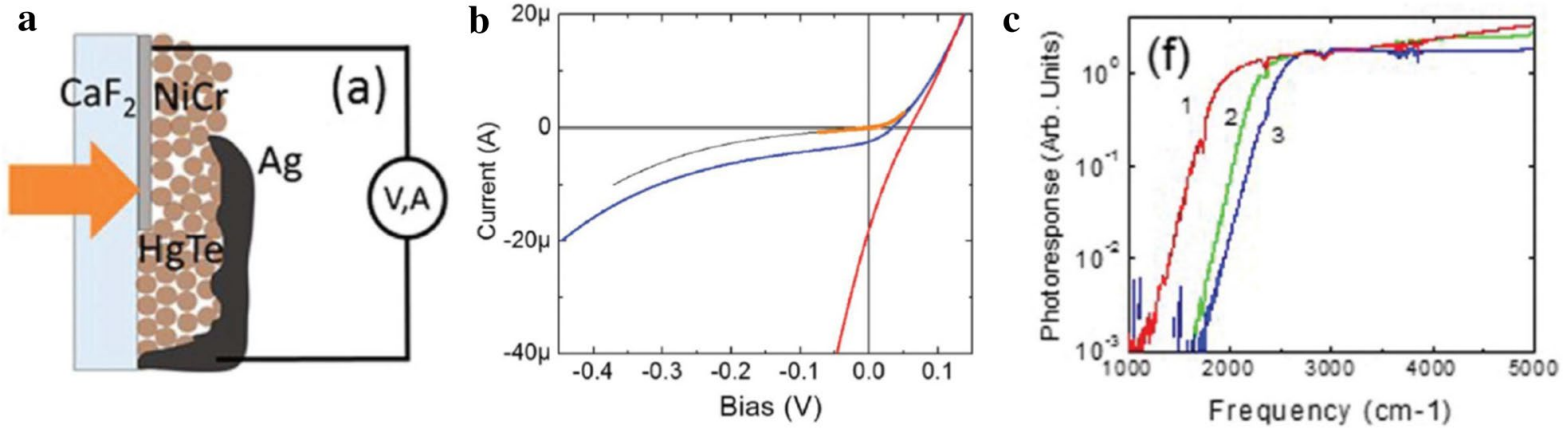
HgTe CQD-based Schottky diodes. **a** A schematic of Schottky diode device structure (CaF_2_/HgTe CQDs/Ag). **b** The device current–voltage characteristics at 90 K. The black and blue curve are the plots obtained with thermal shield closed and open, respectively. The orange curve overlapped with the black plot is the fit to a diode equation with diode ideality factor of 1.26. The red curve is a plot obtained under black body (600 °C) illumination. **c** The spectral photoresponse obtained from HgTe CQD devices having absorption cut-off at 1907 (red, 90 K), 2266 (green, 140 K), and 2500 (blue, 138 K) cm^−1^ (Figures reproduced from ref. [32] with permission. Copyright 2015 AIP Publishing LLC)

The specific detectivity of an HgTe photoconductive device can be greatly improved by reducing the 1/f noise. The 1/f electrical noise component can be minimized if the photodetector operates in zero-bias photovoltaic mode as the dark current approaches zero. A Schottky diode fabricated by depositing Ag top contact on to an ethanedithiol/HCl ligand-exchanged HgTe CQD film (Fig. 19) has been demonstrated to show high response (~ 80 mA/W), attributed to the shorter carrier transport distance compared to that of the previous photoconductive devices, and the absence of 1/f noise, which ultimately led to higher detectivity of 10^10^ Jones at 140 K [32]. In these devices, the possibility that the rectifying junction may have been formed by Ag-diffused p-type HgTe and intrinsic HgTe CQD layers has been discussed. Higher device performance was observed when Ag_2_Te CQD layer was added in between the intrinsic HgTe CQD layer and Ag contact. At 90 K, this device exhibited a detectivity of 4 × 10^10^ Jones with photoresponse cut-off at 5.25 μm. Further analysis revealed that, at this low temperature, the photocurrent fluctuation from ambient background radiation exceeds the intrinsic noise of the device, indicating that the detector has reached background limited infrared performance (BLIP) regime. The rule-07, an empirical relationship that was originally created to be a design rule of thumb for HgCdTe systems has recently become a useful trend for device comparison [68]. The rule describes the dark current density as an exponential function of both cut-off wavelength (λ_c_) and temperature (T) for optimized HgCdTe detectors and is given by the equation J = 8367 × exp[− 1.16·(1.24q)/λ_c_·kT] A/cm^2^. In this context, HgTe CQD photovoltaic devices show a dark current density of 1.3 μA/cm^2^ at 90 K whereas HgCdTe would exhibit similar dark current level at 140 K, indicating that further improvements are needed.

**Fig. 20:**
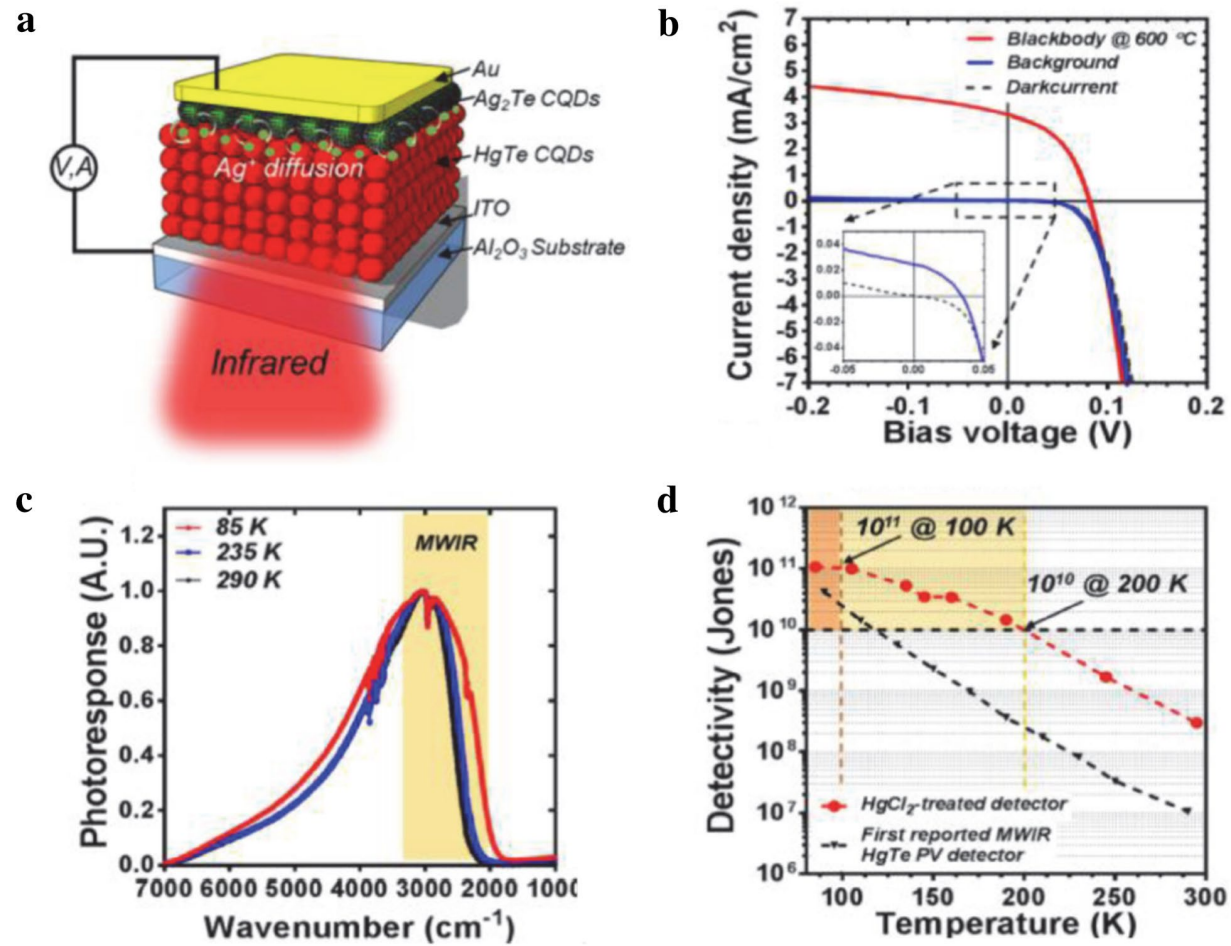
HgTe CQD-based p–n heterojunction devices. **a** A schematic of the device structure. **b** Current density–voltage characteristics obtained at 85 K under dark, background radiation, and black body (600 °C) illumination. **c** The spectral photoresponse obtained at 85, 235, and 290 K. **d** The detectivity plotted as a function of temperature for the current heterojunction device (red) and the first-generation Schottky device (black) (Figures reproduced from ref. [33] with permission. Copyright 2018 American Chemical Society)

**Fig. 21:**
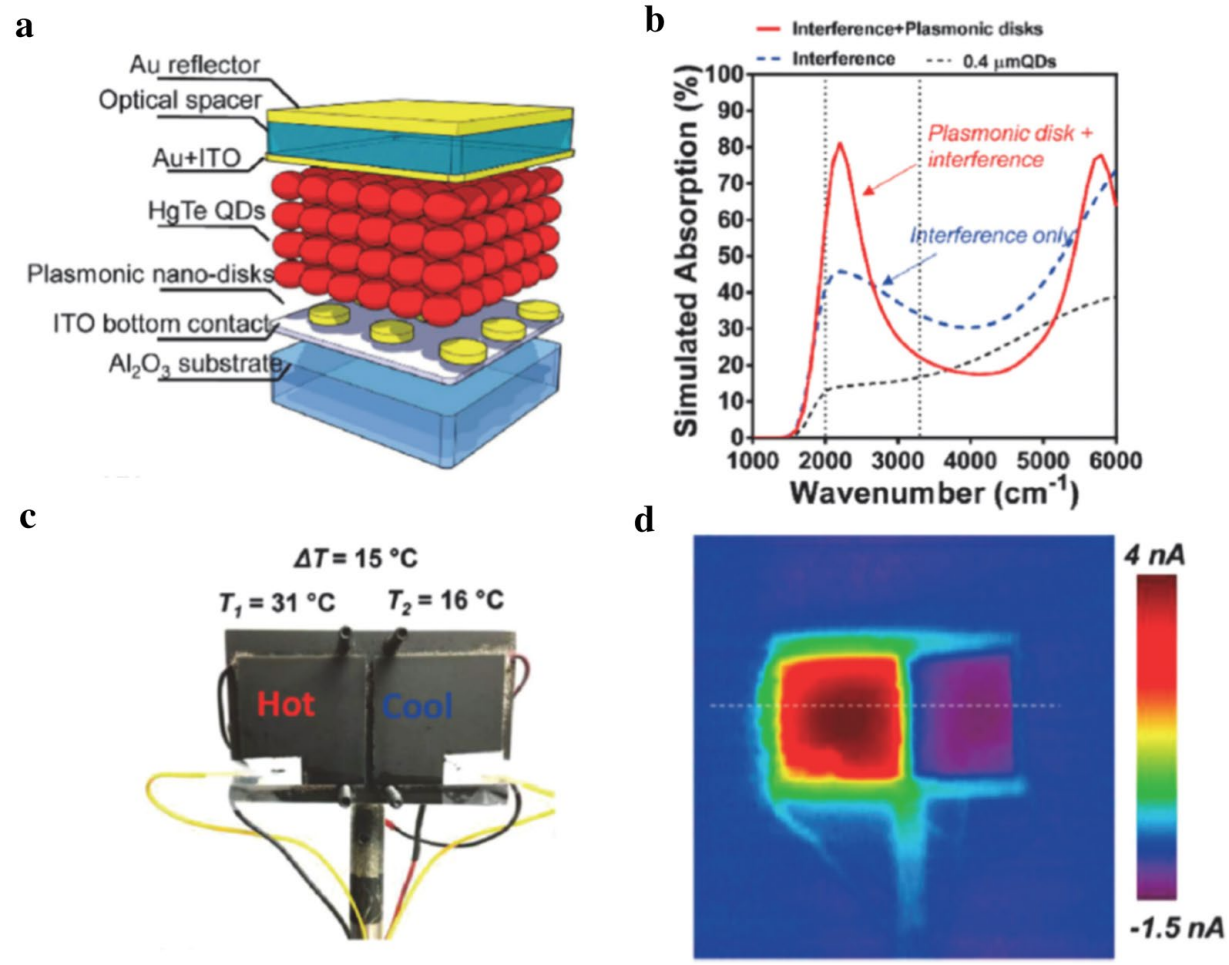
HgTe CQD-based p–n heterojunction device integrated with plasmonic disk array and interference structure. **a** A schematic of the device structure. **b** The spectral response of these devices with and without interference-enhanced plasmonic disc array. **c** Two Peltier coolers mounted on aluminum plates for scanning thermal imaging and NEDT testing. **d** The rainbow-colored imaging data obtained from (**c**) (Figures reproduced from ref. [69] with permission. Copyright 2018 American Chemical Society)

This Schottky diode study has inspired the development of next generation device that is based on p-n heterojunction diode. The device consists of ITO as a bottom contact (illumination side), ethanedithiol/HCl ligand-exchanged thick HgTe CQD layer, HgCl_2_ treated Ag_2_Te CQD inter-layer, thin Ag_2_Te CQD layer, and Au top contact (electrode and back reflector), as shown in Fig. 20 [33]. This device was reported to show detectivity exceeding 10^11^ Jones below 100 K and reached BLIP at 140 K, a significant performance improvement compared to previous generation of devices. At 230 K, a temperature accessible with compact thermoelectric coolers, the detectivity reached 10^9^ Jones which exceeds the performance of microbolometers. The presence of HgCl_2_ treated Ag_2_Te CQD inter-layer has been discussed as a key to obtaining a strong and reproducible rectifying junction. This device has been further enhanced with an interference structure to achieve higher optical absorption and was used to demonstrate scanning thermal imaging with NEDT of 56 mK at 90 K. The design of interference structure requires a top semitransparent contact, which was achieved with 5 nm Au, but simultaneously introduced a large series resistance and resulted in a photocurrent reduction at elevated temperatures. An alternate strategy is to employ plasmonic nanostructures for absorption enhancement. Three main parameters including diameter of the plasmonic discs, HgTe CQD layer thickness, and top Au layer thickness were optimized to enhance the plasmon resonance in MWIR region and to induce optical resonant cavity effect [69]. This approach has led to significant increase in the responsivity which were measured to be up to 1.46 A/W at 4.5 μm resonance wavelength. More importantly, above optical enhancement approaches were found to have minimal effect on the noise level of the device. To date, device that combine two approaches, plasmonic and interference (Fig. 21), demonstrate the best performing device with detectivity of 10^10^ Jones at 220 K and, when used for thermal imaging, the NEDT has been measured to be as low as 14 mK.

### HgSe CQD-based devices

#### Photoconductive devices

**Fig. 22:**
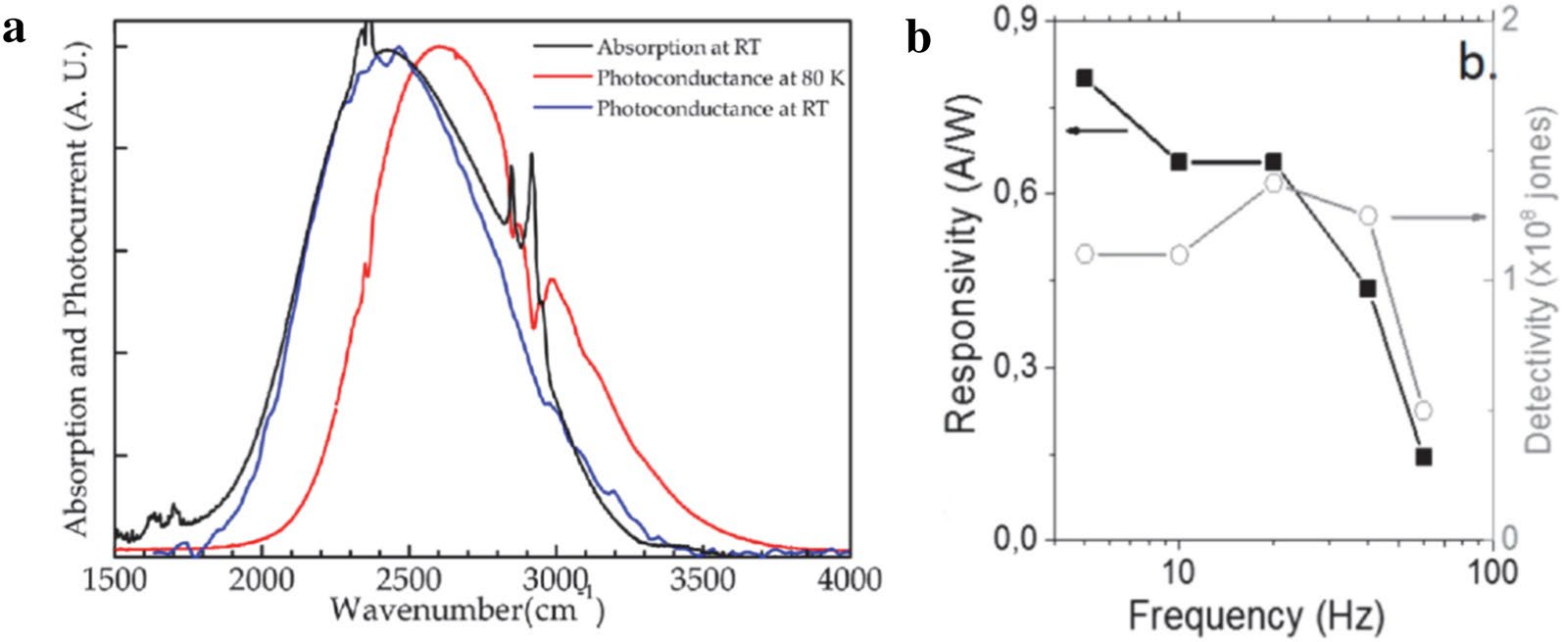
HgSe CQD-based photoconductive devices. **a** Absorption and photocurrent spectra obtained from HgSe CQD films ligand-exchanged with ethanedithiol. **b** A plot of responsivity and detectivity as a function of frequency measured from HgSe CQD films ligand-exchanged with As_2_S_3_-based ligands (Figures reproduced from refs. [42, 44] with permission. Copyright 2016 American Chemical Society)

**Fig. 23:**
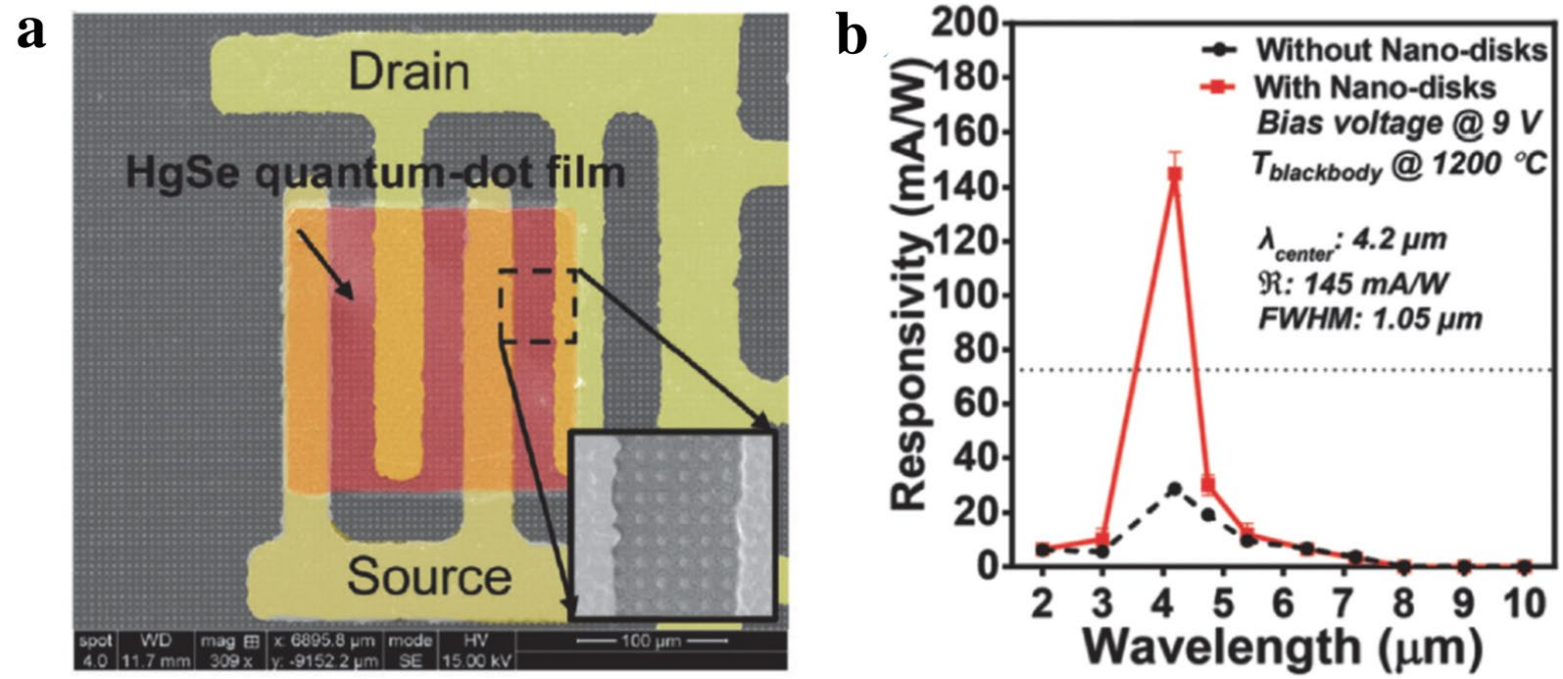
HgSe CQD-based photoconductive device integrated with plasmonic disc arrays. **a** A false color SEM image of the device. **b** The spectral responsivities measured from plasmonic-enhanced HgSe CQD (intraband absorption centered at 4.2 μm) photoconductive devices (Figures reproduced from ref. [51] with permission. Copyright 2017 The Royal Society of Chemistry)

Many CQD-based optoelectronics devices studied so far rely exclusively on the interband optical transition. With the emergence of HgSe CQDs, which exhibit air-stable, n-type self-doping, intraband transition in CQDs has been harnessed for MWIR photodetection. The first demonstration of photoconductive photodetection using intraband HgSe CQD was reported by Guyot-Sionnest group (Fig. 22a) [44]. Using CQD films (intraband absorption peak around 4.4 μm) ligand-exchanged with ethanedithiol, responsivity of 0.38 mA/W and detectivity of 2 × 10^9^ Jones (500 Hz) at 80 K have been measured with a perspective that the precise control of doping would lead to further performance enhancements. Similar to HgTe CQD device development, inorganic ligand exchange approach has been naturally extended to HgSe CQD MWIR devices. The HgSe CQD film ligand-exchanged with As_2_S_3_-based ligand has been reported to maintain n-type doping and exhibit field-effect mobility in the range of 50–100 cm^2^/V s, leading to high responsivity of 0.8 A/W (Fig. 22b) [42]. A specific detectivity of 10^8^ Jones was measured at room temperature, which is comparable to the performance of commercial deuterated triglycine sulfate (DTGS) detectors, with 1/f noise being the dominant noise mechanism. Optimized doping is of upmost importance in realizing high performance devices and has been discussed in various literatures [50, 70, 71] CQD films, after ligand-exchange, typically show modification in carrier concentration which is largely attributed to the bending/shifting in the energy level due to ligand-induced surface dipoles; when the 1S_e_ energy level is lower than O_2_/H_2_O redox potential, water acts as a reducing agent, making CQDs electron-rich in the ambient [50]. This theory is also consistent with the observation that intraband absorbance decreases with decreasing CQD size (stronger quantum confinement and thus, higher energy level position with respect to O_2_/H_2_O redox potential). In devices, having unoptimized doping leads to large dark current. One recent approach to overcome this issue is to utilize HgSe/HgTe core/shell heterostructure CQDs [52]. Introduction of shell is typically known to result in a disappearance of intraband optical absorption [46, 47]. However, properly designed type-II band alignment in HgSe/HgTe structure gives rise to intraband transition when the 1S_e_ state of HgSe core is positioned below the Fermi level of the whole CQD system. The CQD film composed of this heterostructure CQDs showed large reduction in the dark current and stronger temperature dependence in electrical conductivity with activation energy corresponding close to the intraband transition gap. The detectivity at 10 Hz was measured to be enhanced by 30 times compared to that of the device fabricated from HgSe core only CQDs (ethanedithiol ligand-exchanged). The rapid response time (below 50 μs) also confirms that the observed photoresponse are based on optoelectronic mechanism rather that bolometric thermal effects, which has been in question recently. Increasing the optical absorbance by using plasmonic structure, a same path taken in the development of HgTe CQD devices, was demonstrated using HgSe intraband CQDs as well (Fig. 23) [51]. The HgSe CQD film ligand-exchanged with As_2_S_3_-based ligands were integrated with Au plasmonic nanodisks to enhance intraband absorption at 4.2, 6.4, 7.2, and 9.0 μm, which resulted in 517, 288, 257, and 209% improvements, respectively, in their responsivities.

### Ag2Se CQD-based devices

#### Photoconductive devices

**Fig. 24:**
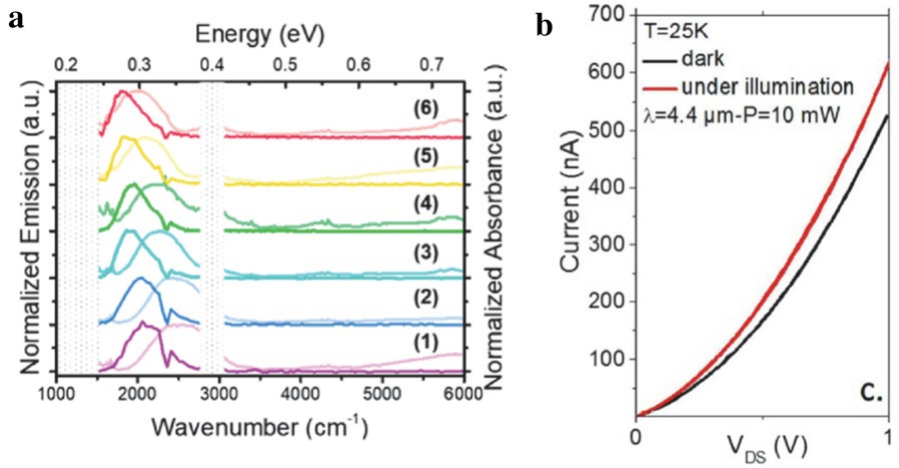
Ag_2_Se CQD-based photoconductive devices. **a** Normalized intraband absorption and emission spectra measured from Ag_2_Se CQDs of various sizes. **b** The change in the current–voltage characteristic of Ag_2_Se CQD film ligand-exchange with ethanedithiol before and after MWIR illumination (Figures reproduced with permission from refs. [58, 59]. Copyright 2018 American Chemical Society)

Intraband absorption in the infrared, in principle, can be achieved with many families of CQD materials under the condition that stable doping can be maintained in the ambient. One recently uncovered, non-toxic alternative to Hg-based CQDs is Ag_2_Se CQDs. It has been studied that CQDs are synthesized in the tetragonal crystal structure, different from bulk, having an excess of silver, which gives rise to excess of electrons in CQDs. The stable presence of excess electrons leads to tunable intraband absorption in MWIR that can be adjusted as a function of CQD size. Recent report of intraband photoluminescence measurements clearly demonstrate that the observed optical transitions are intraband in origin rather than plasmonic (Fig. 24a) [58]. The first photoconductive devices based on Ag_2_Se CQD have been reported very recently. The Ag_2_Se CQD film ligand-exchanged with standard ethanedithiol ligands exhibited limited responsivity, on the order of few μA/W, which is orders of magnitude lower that of the HgSe CQDs (Fig. 24b) [59]. Combined materials and device characterization research efforts are further warranted to examine the full potential of this new colloidal nanomaterial.

## Conclusion and future prospects

In summary, infrared CQDs represent a unique class of material with a potential to bring significant cost disruption in the field of infrared optoelectronics. CQDs exhibiting strong interband optical absorption in the thermal infrared can be produced using inexpensive benchtop colloidal synthesis and mated with existing electronic platforms to complete sensors and imagers with a potential for rapid introduction into commercial markets with low capital investment and low product cost [34]. In addition, emerging intraband CQDs, which are currently confined to photoconductive photodetector demonstrations, may pave the way toward new device concepts and designs.

To date, majority of research has focused on Hg-based CQDs. Mercury in any form is known to be poisonous, majorly affecting neurologic, gastrointestinal and renal organ systems, and poisoning can occur through inhalation, ingestion, and skin absorption [72]. While CQDs incorporated in devices are substrate bound, improper use and disposal of Hg-containing CQD devices could potentially lead to serious health and environmental concerns, especially when devices are deployed on a large scale. In this regard, investigating other libraries of CQDs, such as Ag_2_Se CQDs, is especially important to evaluate a potential alternative to Hg-based CQDs.

Maintaining high detectivity while reducing the cooling requirement has been a collective goal in the infrared research community [73]. In traditional devices, the carrier lifetime shortens due to the onset of Auger process at high temperatures, which necessitates the use of cryogenic coolers for high performance operation [74]. This requirement for stringent cryogenic cooling has been a major impediment to their widespread use in many emerging applications because these coolers are costly to implement, require high input power, and significantly increase the size and weight of the detector. Unlike bulk films where carriers are delocalized, in a CQD film, carrier are localized in a CQD, leading to different carrier interactions and processes. In a CQD, Auger process can occur can either as negative trion, positive trion, or biexciton configuration [75] with Auger lifetime studied to be independent of material, band gap and band structures but follows a universal scaling law with the volume of CQDs [76]. The inter-dot hopping time can also be estimated in CQD films using the Einstein relation [24]. Taking typical mobility of today’s CQD film into account (> 1 cm^2^/V s), hopping time may outpace the Auger lifetime. In this picture, the Auger process can be suppressed since charges rapidly dissociate to neighboring CQDs in a strongly coupled assembly before Auger recombination occurs [77, 78]. Recent reports suggest that CQD films can reach even higher mobility [79, 80] around 20 cm^2^/V s and, most notably, mobility values exceeding 400 cm^2^/V s have also been demonstrated for CQD films with composition-matched inorganic capping ligands [81]. Projecting toward the future, carefully engineered CQD films built-in with Auger suppression may serve as a potential material candidate for realizing long-desired uncooled infrared photodetectors with low size, weight, power consumption and cost (SWAP-C).

## Abbreviations

CQD: colloidal quantum dot
FPA: focal plane array
NIR: near-infrared
MWIR: mid-wavelength infrared
LWIR: long-wavelength infrared
ROIC: read-out integrated circuit
CMOS: complementary metal–oxide–semiconductor
SWIR: short-wavelength infrared
EHP: electron–hole pair
QDIP: quantum dot infrared photodetector
PET: polyethylene terephthalate
NEDT: noise equivalent differential temperature
BLIP: background limited infrared performance
SWAP-C: size, weight, power consumption and cost
TEM: transmission electron microscopy
XRD: X-ray diffraction
PL: photoluminescence
DDT: dodecanethiol
TCE: tetrachloroethylene
EDT: ethanedithiol
ODA: octadecylamine
DDAB: dioctadecyldimethylammonium bromide
FWHM: full width half maximum
TOP: trioctylphosphine
TOP:Te: trioctylphosphine telluride
TMS:Te: (trimethylsilyl) telluride
TBP:Te: tributylphosphine telluride
TFT: thin film transistor
OA: oleic acid
ODE: 1-octadecene
TOPO: trioctylphosphine oxide
